# Multi-Geometry Parameters Optimization of Large-Area Roll-to-Roll Nanoimprint Module Using Grey Relational Analysis and Artificial Neural Network

**DOI:** 10.3390/polym15132909

**Published:** 2023-06-30

**Authors:** Truong Sinh Nguyen, Anton Nailevich Gafurov, Jeongdai Jo, Taik-Min Lee, Seung-Hyun Lee, Kyunghoon Kim

**Affiliations:** 1 Nano-Convergence Manufacturing Systems Research Division, Korea Institute of Machinery and Materials, Daejeon 34103, Republic of Korea; sinhnguyen@kimm.re.kr (T.S.N.); anton@kimm.re.kr (A.N.G.); micro@kimm.re.kr (J.J.); taikmin@kimm.re.kr (T.-M.L.); 2 School of Mechanical Engineering, Sungkyunkwan University, Suwon 16419, Republic of Korea; 3 Department of Nanomechatronics, University of Science and Technology, Daejeon 34113, Republic of Korea

**Keywords:** nanoimprint lithography, roll to roll, force distribution, nip pressure, uniformity, grey relational analysis, artificial neural network

## Abstract

Micro- and nanofabrication on polymer substrate is integral to the development of flexible electronic devices, including touch screens, transparent conductive electrodes, organic photovoltaics, batteries, and wearable devices. The demand for flexible and wearable devices has spurred interest in large-area, high-throughput production methods. Roll-to-roll (R2R) nanoimprint lithography (NIL) is a promising technique for producing nano-scale patterns rapidly and continuously. However, bending in a large-scale R2R system can result in non-uniform force distribution during the imprinting process, which reduces pattern quality. This study investigates the effects of R2R imprinting module geometry parameters on force distribution via simulation, using grey relational analysis to identify optimal parameter levels and ANOVA to determine the percentage of each parameter contribution. The study also investigates the length and force ratio on a backup roller used for bending compensation. The simulation results and the artificial neural network (ANN) model enable the prediction of nip pressure and force distribution non-uniformity along the roller, allowing the selection of the optimal roller geometry and force ratio for minimal non-uniformity on a specific R2R system. An experiment was conducted to validate the simulation results and ANN model.

## 1. Introduction

Currently, the shortage of electronic components is a major challenge for electronics manufacturers around the world. The demand for such components has increased in recent years, as human life has become more dependent on electronic devices [1,2,3]. With the rapid growth of Industry 4.0 and the Internet of Things (IoT), flexible devices have garnered significant attention [4,5,6,7,8,9,10]. This poses a challenge for the electronic components manufacturing industry, particularly for electronic devices based on micro- and nanopatterns.

Among the various micro- and nanofabrication technologies available, imprinting lithography has several notable advantages. It can be performed continuously with very high productivity [11,12,13,14,15], and it can process small patterns down to 10 nm via very simple principles [16,17,18,19]. NIL is considered the most productive nanofabrication method, especially when it can be applied to the R2R system by using a soft mold to replicate the pattern onto the target substrate. Among the various types of R2R NIL, UV R2R NIL is considered the most productive method as it has a fast curing time of just a few seconds [11,12,13].

Despite numerous studies on the R2R NIL system, its implementation on a large scale has not been extensively investigated. Most studies have only been performed on a small region with a processing width of less than 300 mm [20,21,22,23,24,25]. Through mathematical modeling and simulation, Aarts et al. demonstrated the influence of roller deformation on the force distribution in the contact area of a paper-processing system, even for an area of only 300 mm [26]. It is apparent that if R2R NIL performs on a larger scale, the roller deformation issue will become more severe and this issue should be taken into consideration.

In all applications that use an R2R system, the distribution of force along the contact area is of utmost importance. Non-uniform force distribution can affect web tension and cause contraction and wrinkling of the web, leading to defects on micro-/nanopatterns or the paper surface in R2R paper processing [27,28,29]. In imprinting lithography, for high-aspect-ratio patterns, it is crucial to effectively control the adhesion and friction between materials in order to achieve a successful pattern transfer and ensure the reliability. Numerous theoretical and experimental studies have been conducted to investigate the tribological phenomena in NIL and mitigate defects caused by adhesion and friction [30,31,32,33]. Particularly, a high force is required to reduce the residual layer thickness and ensure that the resin can be filled into the deep micro- or nanochannel [34,35]. In the calendaring processing of battery manufacturing, the nip pressure between the two rollers also needs to be well and evenly controlled to control the porous structure of the electrode coating layer [36,37].

Therefore, surface roughness and geometric tolerance for rollers demand strict concentricity, cylindricity, and parallelism during assembly [38]. Normally, when two rollers come into contact with each other with a specific force, the applied force tends to concentrate at the two ends and gradually decrease towards the center. Therefore, geometric shape adjustments have been proposed to eliminate this phenomenon, namely, the crowned roller. In this case, the diameter of the roller to be machined is smaller at both ends and larger at the center [39,40,41,42].

Recently, a few studies have addressed the bending problem that occurs in R2R systems when operating in a large area, and several methods have been proposed to compensate for the deformation of the roller system, such as the use of one or more additional rollers to eliminate bending [43,44]. However, these studies only deal with specific cases from the authors’ research, and the results cannot be applied to many different cases, as the R2R system varies in size in terms of geometry and processing parameters. Therefore, in this study, we will investigate in detail the influence of geometrical parameters on the force distribution along the contact area, where uniformity is a crucial factor in most applications that utilize the R2R system.

The rapid development of computers makes the simulation of systems faster and more flexible than ever. Realistic models can be predicted through simulation without spending too much effort and money. Therefore, in this study, a series of simulations will be performed on the conventional R2R system as well as the system with the support of an additional module for deformation compensation. The simulation results will be collected and analyzed using various methods to determine the influence of the parameters. In particular, we will use an ANN model to learn simulation data and predict the force distribution and non-uniformity at the processing area for a specific R2R system. The results will allow us to evaluate the processing ability of the R2R system and choose the appropriate parameters for the additional roller used for compensating the bending. Specifically, we can predict the optimal length and force ratio for the backup roller to enhance the conventional R2R system.

## 2. Finite Element Modeling (FEM) of Imprinting Module

**Figure 1:**
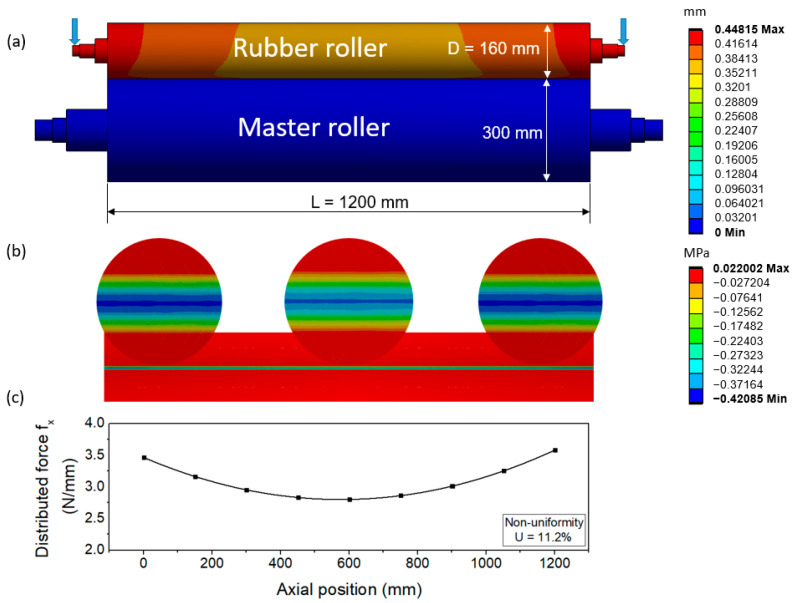
3D full model of R2R module with deformation of rubber roller (**a**) pressure distribution (**b**) and distributed force (**c**) on contact area on large scale R2R NIL system; f = 3 N/mm.

To confirm the deformation of the roller, we employed the commercial finite element analysis (FEA) software, ANSYS Mechanical. The master roller and rubber roller were assumed to be deformable bodies, and all bodies were modeled as homogeneous and isotropic bodies using tetrahedral meshing elements. The processing substrate, as well as micro-/nanopatterns between the two rollers, is considered to have a minor effect, so it is skipped in our model. The steel and rubber properties are shown in Table S1. The 3D model with deflection when applying force is shown in Figure 1a. In this study, instead of using the total force applied to the rubber roller, we will use the average applied force f which represents the applied force over a length of 1 mm of the roller. This ensures that the force applied per unit length remains constant when changing the length of the roller. For instance, in this simulation scenario where a force of 3600 N is applied to a 1200 mm roller, we have f = 3 N/mm. The deflection of the imprinting module is shown in Figure 1a, with deflection mainly concentrated on the rubber roller. It can be seen that the master roller is made of steel and has a much larger diameter than the rubber roller, so the deformation of the master roller is almost negligible. In Figure 1b, the contact area between the two rollers shown the distributed pressure focused on both ends and getting smaller towards the middle.

From the simulation, the pressure distribution data of all elements on the contact areas of the two rollers were obtained as a matrix, with each row representing the nip pressure distribution at that point. From each distribution, we obtained the distributed force at one specific point. Repeating the procedure for all distributions yielded the distributed force along the roller, as shown in Figure 1c. From the force distribution, non-uniformity U which represents the difference in the distributed force on the contact area along the roller can be defined by Equation (1) as:

(1) $$U=\frac{\left.{\mathrm{Max}(F}_{\max}-{\;F}_{\mathrm{mean}}{,\;F}_{\mathrm{mean}}-{\;F}_{\min}\right)}{F_{\mathrm{mean}}}\times \;100\%$$

where F_max_, F_min_, and F_mean_ are the maximum, minimum, and the average values of the distributed force (N/mm) along the roller, respectively.

**Figure 2:**
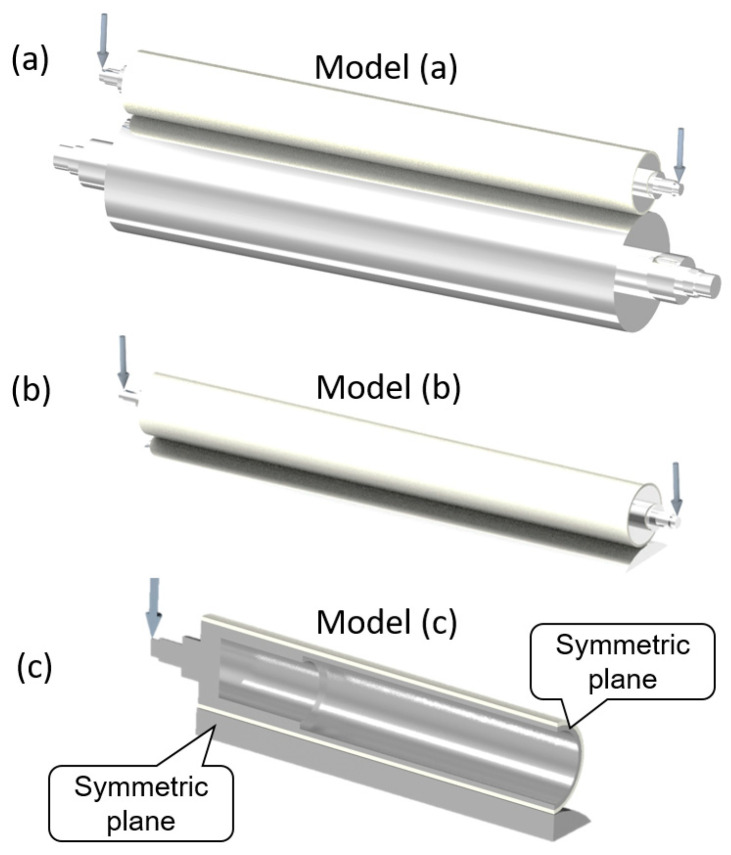
Full 3D model (**a**), model with simplified master roller (**b**), and model with symmetric conditions (**c**).

To investigate forced uniformity depending on roller geometry, a series of simulations must be performed. For an accurate simulation of the imprinting module on a large scale, the element size should be as small as possible. However, this increases the computation time significantly. For example, the minimal mesh size for a 1200 mm roller length is 0.5 mm at the contact area, which requires 7,328,300 nodes and 5,061,504 elements. Such a large number of nodes and elements leads to a simulation time of several hours for each case. As mentioned, the master roller deformation is almost zero, so a simplified model of the master roller was made as in Figure 2b. Additionally, as the model is symmetric, a simplified model with symmetric conditions can be used to significantly reduce the computation time, as shown in Figure 2c. The process of model simplification is described in Figure 2, and the statistics of nodes, elements, and computation time for each case are shown in Table S2.

**Figure 3:**
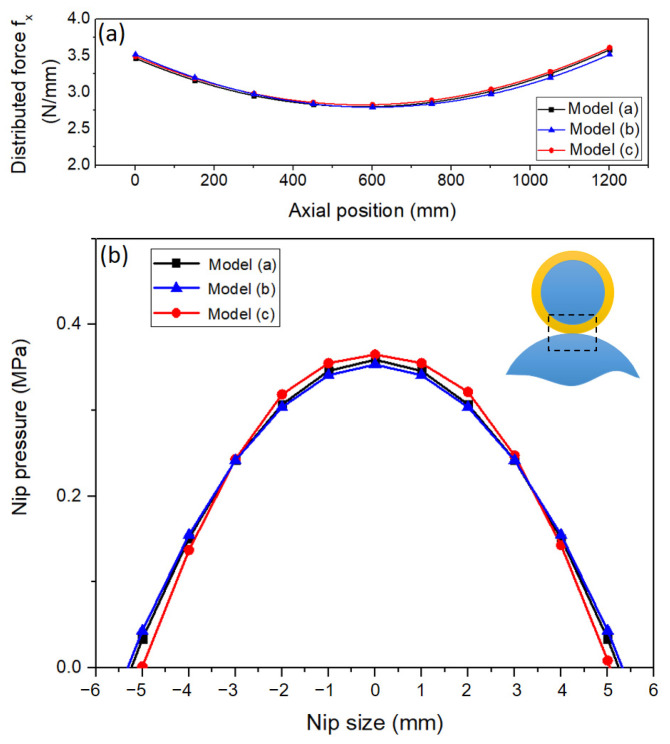
Force distribution along the roller (**a**). Nip pressure on the nip size at the middle of roller of several models (**b**).

The simulation result comparison of the full model and simplified models is shown in Figure 3. The obtained distributed force along the roller as well as nip pressure at the middle of the roller are almost the same for the three cases. Although there is still a slight deviation between the models due to simplifying the master roller of model (b) and the application of symmetric conditions model (c) causing the elements at the symmetrical position to be not as continuous as on the full model, the deviations have no significant effect on the force distribution curve along the roller. Therefore, the simplified model in Figure 2c can be used for simulation to save computing time.

Another factor that can impact force distribution is the axis diameter. To investigate its effect on non-uniformity, various values of the axis diameter were modeled, ranging from a small value of 20 mm to a larger value of 60 mm. A larger axis diameter improves the rigidity of the roller. However, if the size is too large, it may not be necessary, which would increase material costs and result in a cumbersome system. On the other hand, if the axis diameter is too small, the probability of failure is high at the position of the highest stress concentration, as shown in Figure S1. The effect of axis diameter on non-uniformity is shown in Table S3.

Simulation results show that increasing the diameter from 20 mm to 60 mm (a three-fold increase) only improves non-uniformity by 2%. Therefore, the axis diameter has only a minimal effect on force distribution. To ensure the system’s durability and robustness, a middle value was chosen. In our simulations, we kept the axis diameter constant at 40 mm to simplify the simulation work and facilitate the investigation of the main affected parameters.

## 3. Effect of Rubber Roller’s Geometry on Non-Uniformity

To enhance manufacturing performance, it is recommended to make the roller as long as possible. In this study, we investigated several geometry parameters of the roller on the imprinting module. This module is the most crucial part of the R2R NIL system, as it is responsible for replicating the pattern on a soft mold on the master roller to the substrate.

To analyze the effect of rubber roller geometry to non-uniformity, Taguchi’s design of experiment and grey relational analysis were used [45,46]. Taguchi’s design of experiment offers a simple and systematic approach to optimize the simulation cases together with grey relational analysis which is suitable for solving problems with multiple factors to find the optimal parameters for process [47,48,49,50]. Finally, the analysis of variance (ANOVA) [51,52] for grey relational grade is used to find out which controllable factors have significantly affects the output.

The simulation was conducted using an L9 standard orthogonal array design with three input parameters: rubber roller diameter D, rubber roller thickness T, and rubber roller length L. Each factor was designed with three levels corresponding to small, medium, and large levels of roller geometry. Based on practical experience and simulation results, we applied the hypothesis of ignoring the bending of the master roller and used a simplified model with symmetric conditions for the simulation. Table 1 shows the geometry parameters and their respective levels, and geometry details are shown in Figure S2. Typically, the roller length in a popular R2R system ranges from 300 mm to 600 mm. However, this study focuses only on large-area R2R systems, so we chose a roller length ranging from 800 mm to 1600 mm. This range was expected to show a clear deviation of pressure distribution for data analysis. For the diameter and thickness of the rubber roller, we selected a range of 130 mm to 190 mm and 9 mm to 15 mm, respectively, based on our current R2R systems and practical experiments. The simulations were conducted using the L9 standard orthogonal array design, and the simulation results are included in Table 2 and shown in Table S4.

Next, grey relational analysis was used to obtain the optimal condition. In our study, we only had one output value, which is non-uniformity, making the analysis process relatively simple. The calculations from the obtained data are shown in Table 2.

The non-uniformity (U) value was normalized between 0 and 1. Since the non-uniformity characteristic is such that higher values indicate worse imprinting quality, it is considered a “smaller-the-better” performance characteristic. Thus, the normalized data can be expressed using the following equation:

(2) $$x_{i}=\frac{\max\left(U_{i}\right)-U_{i}}{\max\left(U_{i}\right)-{\min(U}_{i})}$$

From the normalized data, x_i_ = 1 is considered the best case when U reaches the minimal value. Since our study only investigates one output (non-uniformity), the grey relational grade (GRG) is equal to the grey relational coefficient, which is used to determine the deviation between x_i_ and GRG. The larger the grey relational coefficient, the closer x_i_ is to the optimal case.

The deviation sequence of the reference sequence is given by: ${∆}_{i}=\left|x_{o}-x_{i}\right|$ with $x_{0\;}=1$ (the best normalized data of U); ${∆}_{\min}=\min\left\{{∆}_{i}\right\}$, ${∆}_{\max}=\max\left\{{∆}_{i}\right\}$. The distinguishing coefficient ε∈(0,1] is used to expand or compress the range of the grey relational coefficient and may be adjusted based on practical needs of the system [47]. The grey order is always same no matter what the distinguishing coefficient is. In this study, ε is assumed to be 0.5.

The GRG is calculated using the following equation:

(3) $${\mathrm{GRG}}_{i}=\frac{{∆}_{\min}+\varepsilon {∆}_{\max}}{{∆}_{i}+\varepsilon {∆}_{\max}}$$

**Figure 4:**
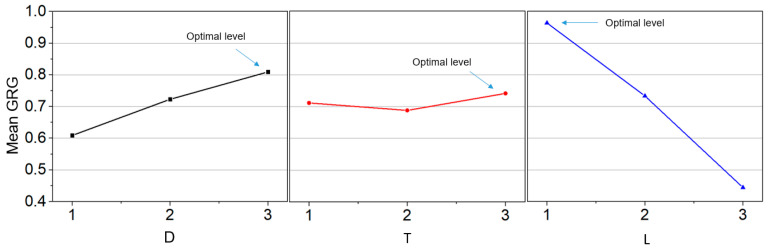
Grey relational grade graph.

The mean of the grey relational grade for each level geometry parameter were calculated and are graphed in Table 3 and Figure 4.

The larger value shown in Table 3 (bold) for each parameter mentions that the lower U can be obtained with the corresponding level. From the graph, the higher the grey relational grade, the better uniformity of imprinting pattern can be obtained.

Therefore, we can obtain optimal uniformity when using the geometry parameters with 190 mm diameter, 15 mm thickness, and 800 mm of length. This is completely understandable when the uniformity reaches the optimal value when processing with rubber roller with the largest diameter (D level 3), the largest thickness (T level 3), and the smallest length (L level 1).

To verify the analysis result, the simulation was implemented with these optimal parameters, which resulted in a non-uniformity of U = 1.8414%. Compared to the best case of the testing data (case #8 with U = 2.0479%), the uniformity improved by 10%.

In the next step, the analysis of variance (ANOVA) was used to quantify the impact of the geometry parameters on the non-uniformity based on the grey relational grade (GRG) obtained in Table 2. The results are shown in Table 4 below with R-sq = 98.44%. The contribution of each geometry parameter can be calculated using the sum of the squares of each parameter in the total sum of squares. The ANOVA related equations are shown in Appendix S1 (Supplementary S1).

Based on the ANOVA for GRG, the rubber roller length significantly affects the pressure uniformity at the contact area (84.87%), followed by the roller diameter (12.66%), while the rubber roller thickness has minimal impact (0.9%). Therefore, to enhance process quality, it is not recommended to increase the thickness of the roller. Instead, the process can be improved by increasing the diameter of the roller. Evidently, decreasing the length of the roller is impossible because it is related to the target imprinting area. For large-area processing, it is necessary to use some method to compensate the deformation.

## 4. Non-Uniformity Prediction for Conventional R2R Imprinting Module

**Figure 5:**
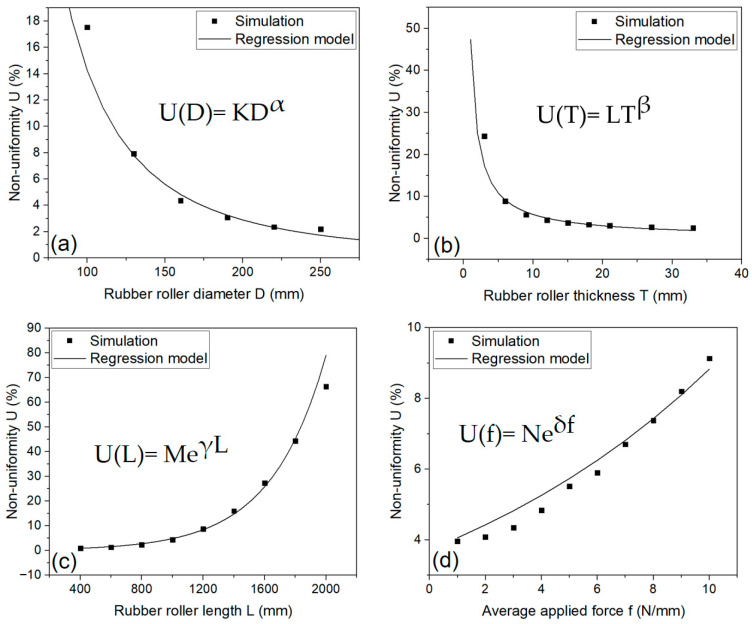
Non-uniformity U versus D when T = 12 mm, L = 1200 mm, f = 3 N/mm (**a**); Non-uniformity U versus T when D = 160 mm, L = 1200 mm, f = 3 N/mm (**b**); Non-uniformity U versus L when D = 160 mm, T = 12 mm, f = 3 N/mm (**c**); Non-uniformity U versus f when D = 160 mm, T = 12 mm, L = 1200 mm (**d**).

In addition to the collected simulation data, we performed extensive simulations to expand the range of geometry parameters from very low to high magnitudes while keeping the other parameters constant, surveying the trend of non-uniformity and establishing a regression model from the simulation data. This approach enables the prediction of non-uniformity for any R2R system from geometry information. Besides the geometry parameters, we also investigated the effect of force. Increasing force causes the imprinting uniformity to become worse. The simulation results, as well as the fitting curve with an approximate equation, are plotted in Figure 5.

From Figure 5a, when the simulations were performed with diameters ranging from 150 mm to 250 mm, the non-uniformity only ranged from 2% to 4%. U only increases dramatically when the diameter is reduced to a size that is too small at 100 mm. In Figure 5b, we can see that when the thickness varies from 10 mm to nearly 35 mm, U almost remains at 5%, and the U value only suddenly increases when the thickness is too thin, <5 mm; with such a thin thickness, the structure of the roller is no longer guaranteed. Figure 5c shows that U has a significant increase when changing in length. Particularly, as the roller length increases from 1200 mm to 2000 mm, there is a notable increase in U from 10% to nearly 65%. These results are in full agreement with the previous grey analysis. In this regression model, the average applied force was also included in Figure 5d, because this is also a factor that changes the uniformity during processing. Therefore, f ranges from 1 N/mm to 10 N/mm were evaluated to obtain a more accurate prediction model. From the trend line of each graph, we can establish the non-uniformity prediction Equation (4) as:

(4) $${U=\mathrm{ED}}^{\alpha }T^{\beta }e^{\gamma L}e^{\delta f}$$

where E = K × L × M × N.

Take the natural logarithm of both sides:

$${\mathrm{lnU}=\ln(\mathrm{ED}}^{\alpha }T^{\beta }e^{\gamma L}e^{\delta f})=\ln\left(E\right)+\alpha \ln\left(D\right)+\beta \ln\left(T\right)+\gamma L+\delta f$$

Linear regression was applied with the final equation.

The regression coefficients were obtained: α=−2.30891, β=−0.92891, γ=0.00276, and δ=0.08412, and the intercept $\ln\left(E\right)=7.99403$.

The final non-uniformity prediction equation was obtained as Equation (5) below:

(5) $$U=2963.21D^{-2.30891}T^{-0.92891}e^{0.00276L}e^{0.08412f}$$

The model accuracy measure via the mean square error MSE=0.00677.

In short, from the regression model, the non-uniformity of the large-area conventional R2R NIL can be predicted using the geometry features and applied force.

## 5. Non-Uniformity and Distributed Force Curve Prediction for R2R Imprinting Module with Backup Roller

### 5.1. R2R Imprinting Module with Backup Roller Simulation

**Figure 6:**
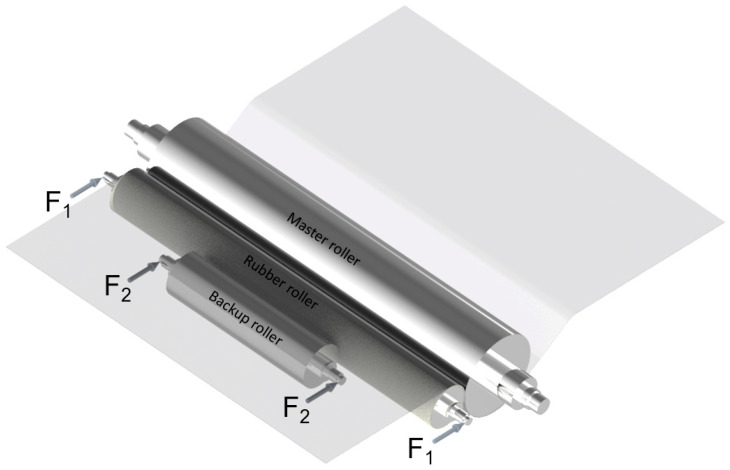
R2R imprinting module with backup roller.

Figure 5c shows that when the length increases to 1200 mm, the non-uniformity U increases to higher than 10% and becomes even worse at 1600 mm and 2000 mm with U values of 27% and 62%, respectively. In such cases, a method to compensate for the R2R module deformation is necessary, which is described in Figure 6 with a backup roller acted upon by a force 2F_2_. The average distributed force f on the contact area was kept constant at 3 N/mm for all simulation cases, and the applied force parameters are given as ΣF = 2F_1_ + 2F_2_ and force ratio R = F_2_/F_1_.

With this proposed model, the length of the rubber roller and backup roller was surveyed from 1200 mm to 2000 mm and 200 mm to 1000 mm, respectively. The detailed geometry of the backup roller is shown in Figure S3. The force ratio was applied from 1 to 9. Five levels for each parameter were used for simulations. The full factorial design of experiments gave 125 combinations of three input variables as shown in Table 5, and the simulation results are shown in Table S5.

**Figure 7:**
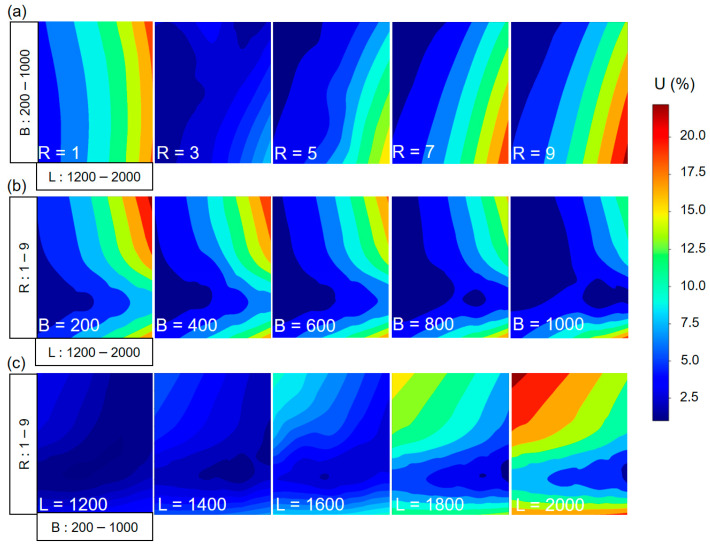
Contour plot for non-uniformity: (**a**) rubber roller length L vs. backup roller length B; (**b**) rubber roller length L vs. force ratio R; (**c**) backup roller length B vs. force ratio R.

From the dataset, the 2D contour of the output value can be plotted in Figure 7. From the contour, the relationship between two variables and non-uniformity can be shown.

Figure 7a shows the contour plot for non-uniformity when R values are kept constant from 1 to 9, respectively. It is easy to see that R = 3 results in the best U for all L and B values. When R = 1, it can be observed that U increases uniformly with the increase in L, indicating that the backup roller has little impact on U. In other words, when R = 1, the force acting on the backup roller is not large enough to compensate for the bending of the rubber roller, especially with rollers longer than 1600 mm. In the other cases, the contour of U tends to lean to the right and to be maximum in the bottom right area, where L is large and B is small. This implies that the length of the backup roller needs to increase linearly with the length of the rubber roller to achieve the best uniformity. This is confirmed in Figure 7b; as the length of the backup roller increases, the area of good non-uniformity also expands. This expansion is more prominent in the R values ranging from 5 to 9. Therefore, when using a longer backup roller, an increase in R should be taken into consideration. Figure 7c shows that as L increases, the selection area for parameters to have a good U becomes smaller. Therefore, choosing the right backup roller length and force ratio is essential in large-area R2R systems.

### 5.2. Artificial Neural Network for Non-Uniformity Prediction

The sequential model was used with a feedforward backpropagation algorithm. The dataset consisting of 125 cases was divided into 75% training data and 25% testing data using radom_state = 42, this ensures that the dataset will be split in the same way every time the code is executed. In a preprocessing step, the input values were scaled using the standard scaler before training. In ANN model, the number of input and output neurons in the first and final layer is equal to the number of factors and outputs in the dataset. Simultaneously, there is no specific rule for choosing the batch size as well as the architecture of the neural network. The TensorFlow and Keras frameworks in Python were used for programming. The model parameters were determined empirically and summarized in Table 6.

**Figure 8:**
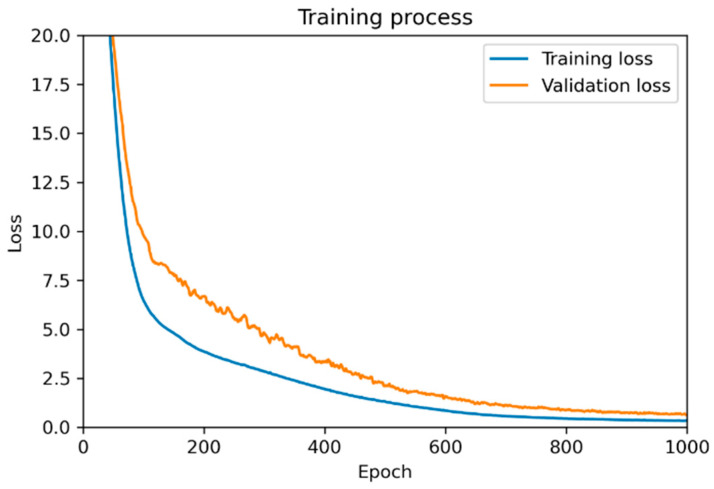
Data training process by ANN.

The training process is shown in Figure 8 with 1000 epochs. The training loss is a measure of how well the model fits the training data. It is calculated based on the discrepancy between the predicted output of the model and the target value (simulation value). The validation loss is calculated in a similar way to the training loss but using the testing dataset. It can be seen with 1000 epochs that both training loss and validation loss gradually decrease and stay constant as the model learns to make better predictions and generalize unseen data.

**Figure 9:**
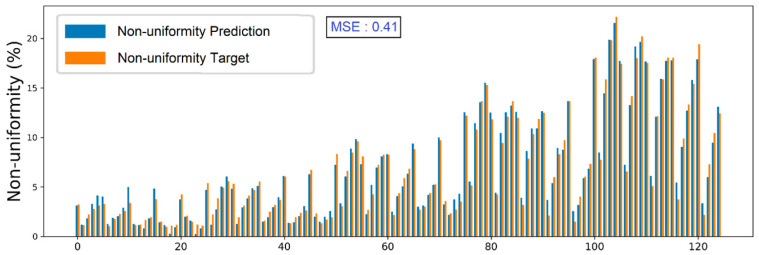
Comparison between prediction and target data non-uniformity for all cases.

The prediction data for 125 cases including training data and validation data were plotted in Figure 9.

By using an ANN model, the non-uniformity of a large-area R2R system can be predicted by inputting the rubber roller length, backup roller length, and the force ratio applied on those rollers with MSE = 0.41. It is considered a good value for predicting non-uniformity. The comparison between prediction and target values shown in Figure 9 is quite consistent for most of the cases including training and validation cases.

### 5.3. Artificial Neural Network for Distributed Force Curve Prediction

In Section 5.2, the prediction of non-uniformity can be made by considering the following inputs: the length of the rubber roller and backup roller, and the force ratio applied on both rollers. However, the effect of changing the average applied force f was not considered, and it was assumed to be constant at 3 N/mm. In this section, the force level will be added as another input, and the impact of varying the average applied force level from 1 N/mm to 7 N/mm will be investigated, as shown in Table 7.

**Figure 10:**
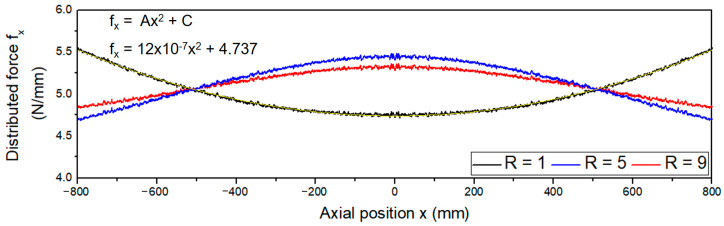
Distributed curve with various force ratio R with f = 5 N/mm, L = 1600 mm, and B = 600 mm.

Similarly, the full factorial design of experiments resulted in 108 combinations of 4 input variables. The desired output in this part is the distributed force curve along the roller. As per the result in Figure 1c, the distributed force along the axis is represented by of a quadratic equation $f_{x}{=\mathrm{Ax}}^{2}+C$. For each simulation case, when keeping average applied force f, roller length factors and changing the force ratio level from low to high (1~9), the curve will be changed from a convex to a concave shape, as illustrated in Figure 10. Each curve was fitted with a quadratic equation; the coefficient A and the intercept C were collected as the outputs of the dataset for all simulation cases. The dataset for the ANN model to predict the distribution curve of the R2R imprinting module on a large scale when using the backup roller is shown in Table S6, and the parameters of the ANN model are provided in Table S7.

In this model, several constant learning rates were used, but the results show that there was a significant fluctuation during the training process, as shown in Figure S4a. Therefore, a learning rate with an exponential decay of 0.99 was used. The basic idea of exponential decay learning rate is to gradually reduce the learning rate over time as the training progresses, allowing for smaller steps toward the optimal solution. The performance of the latter model is better, with less fluctuation, as shown in Figure S4b.

**Figure 11:**
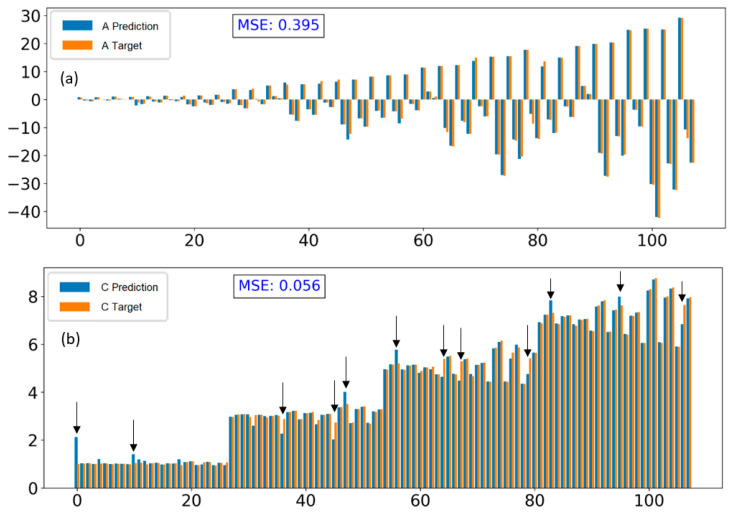
Comparison between prediction and target data of A (**a**) and C (**b**) for all cases with large deviation cases pointed out using learning rate of 0.025 and an exponential decay 0.99.

The comparison between the prediction data and target data includes coefficient A and intercept C for all cases (training data and validation data) with different learning rate strategies, shown in Figure 11 and Figure S5. The results show that the performance of the model is improved when using learning rate with exponential decay. The MSE slightly decreases from 0.435 to 0.395 for prediction A and drops from 0.138 to 0.056 for prediction C.

Generally, this model is capable of predicting the force distribution curve in terms of the bending direction, bending level, and the y-intercept indicated by the sign of A and magnitude of A and C, respectively. However, upon closer inspection of the intercept C, there are still deviations in some cases, which are pointed out in Figure 11b with arrows. Particularly, when all the distribution curves between the prediction and target data are plotted and compared, as shown in Supplementary S2, the results indicate that most cases are consistent in terms of bending level and bending direction, indicating that the prediction of A is accurate. However, the deviation of vertical translation or shift of the parabola along the y-axis is still significant in some cases. This error is also demonstrated in Figure S4b through the loss function, which shows a gap between the training loss and validation loss. 

**Figure 12:**
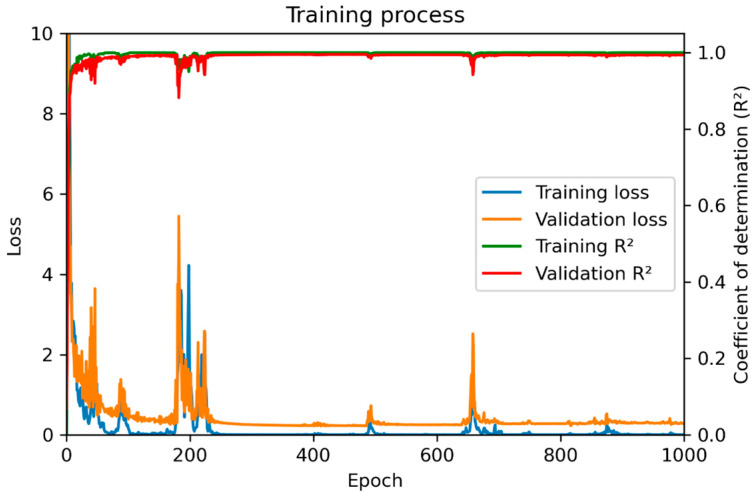
The training process of the model with the residual error of intercept C instead of C (learning rate 0.025 with exponential decay 0.99).

To address this issue, a closer examination of the dataset features was conducted. It can be seen that there is a one-to-one correlation between C and the average applied force f which means that f has a major impact on C while the other parameters have a minor impact on it. Therefore, choosing residual error (obtained by subtracting f from C) instead of C helps increase the sensitivity of the effects of other parameters on C and reduce the error in the prediction result because the final prediction of C is calculated based on f and the predicted residual error. From the argument above, the dataset was modified and the performance of the model is shown in Figure 12, which improves when the validation loss becomes smaller compared to the previous model’s performance (Figure S4b).

**Figure 13:**
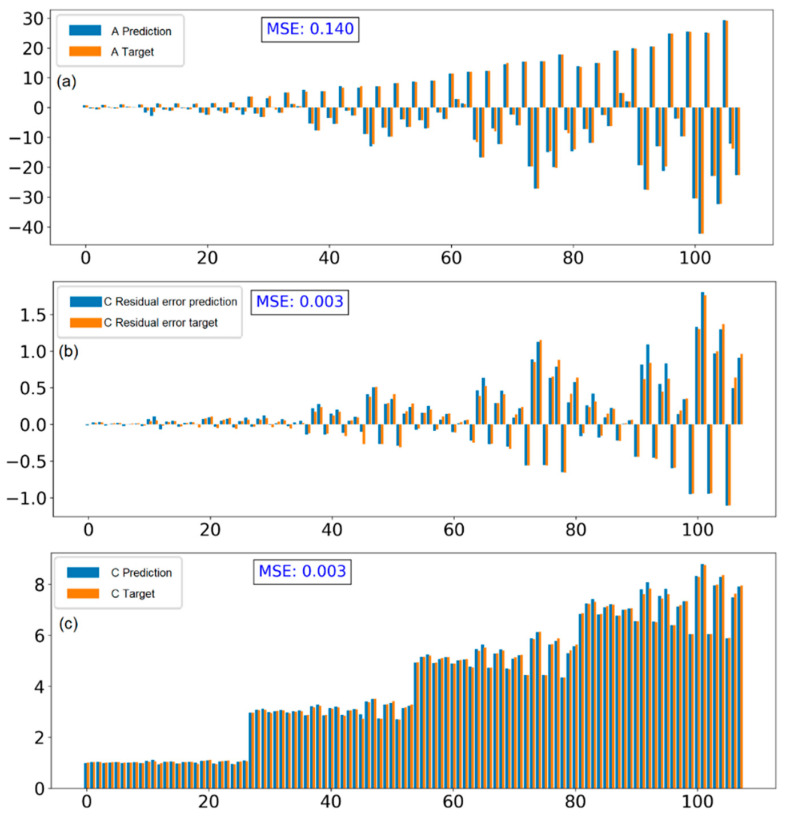
Comparison between prediction and target data of coefficient A (**a**), residual error of intercept C (**b**), and intercept C (**c**) after re-calculation based on average applied force f with learning rate 0.025 and exponential decay 0.99.

By using this model, the MSE between prediction and target data improved. The MSE has not only improved for C prediction results but also coefficient A, namely, it has decreased significantly from 0.395 to 0.14 and 0.056 to 0.003 for A and C prediction, respectively. The comparison results are shown in Figure 11 and Figure 13.

With the improved model, the prediction results are consistent with the target data, and there are no longer cases of large errors when predicting intercept C. This proves that by taking into account the linear relationship between applied force and intercept C, the model performance has significantly improved. The prediction results for all cases, including both training and validation data, are plotted in Supplementary S3. Compared to the results of the previous model, the majority of the force distribution curves have been correctly predicted in terms of bending direction, bending level, and vertical translation

## 6. Experiment

**Figure 14:**
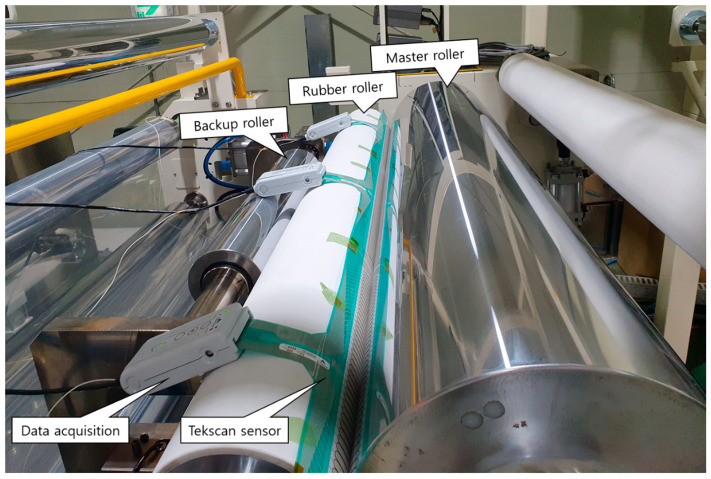
Setup of nip pressure measurement by Tekscan sensor on R2R module.

To verify the simulation result as well as the ANN prediction model, the measurement of the pressure distribution on the contact area of our current R2R NIL system, which has a 1400 mm rubber roller and a 600 mm backup roller, was performed using a piezoresistive thin-film array sensor (Model 5555, Tekscan, Inc., South Boston, MA, USA). The parameters of the sensor are provided in Figure S6; it has a sensing area of 457 × 55.9 mm that consists of 52 columns, and each column contains 44 sensors. The experimental process was set up as in Figure 14; there were three sensors placed along the contact area between the rubber roller and the steel master roller, and the pressure signal at the contact area was received by the sensor and processed by the I-scan software (Tekscan Inc., South Boston, MA, USA) through the data acquisition electronics device. We experimented by applying the force on both ends of rubber roller and backup roller with the force ratio R = 1, R = 3, and R = 6. The actual force can be controlled by the load cells placed at both ends of rubber roller and backup roller. The input force was tweaked until the load cells reached the magnitude that aligned with our desired force ratio R.

**Figure 15:**
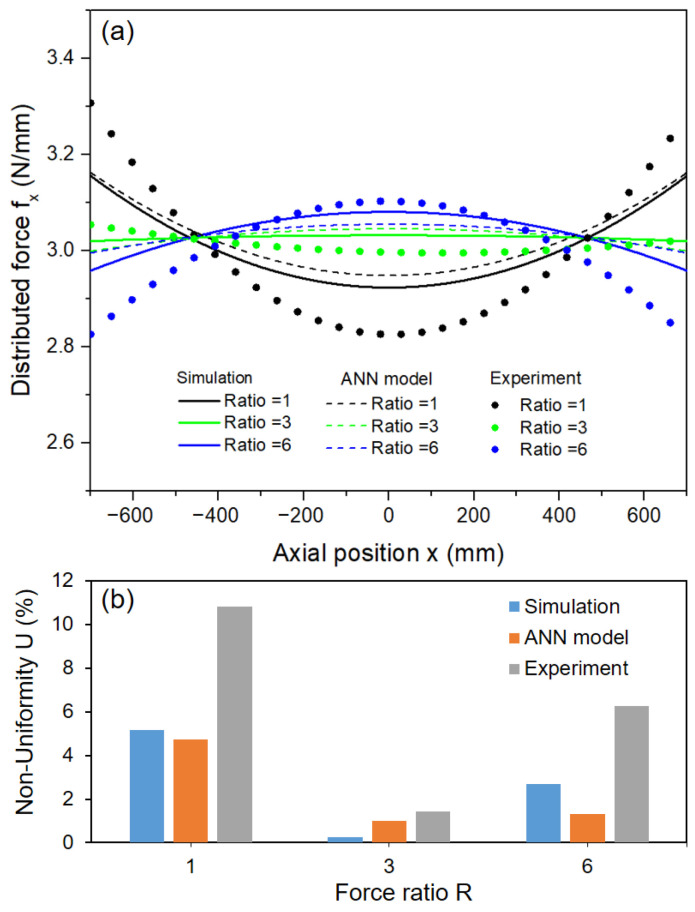
Comparison of force distribution (**a**) and non-uniformity (**b**) between simulation, ANN model, and experiment with f = 3 N/mm, L = 1400 mm, B = 600 mm.

Similar to the procedure for collecting pressure data from the contact area in the simulation part, the pressure data obtained along the roller was gathered as a matrix of pressure values from each sensor. From this data, the distributed force can be calculated at each point along the roller, and the force distribution curve can be obtained. Figure 15a shows the comparison of the force distribution curves obtained by simulation, ANN model, and nip pressure mapping experiment; the corresponding non-uniformity is shown in Figure 15b. 

The error still occurs between the experiment and the simulation, as well as the ANN model. There are several potential factors that can contribute to this issue, including the assumptions made during the simulation process and discrepancies between the material properties used in the model and the actual system. Frictional forces between the parts occurred during the experiment. Errors can also arise from the force acting mechanisms via the air pressure cylinder, and although efforts have been made to minimize these errors through load cell control, complete elimination is not achievable. Finally, there is a potential for errors to arise during the measurement process, including calibration error, sensor alignment process on contact area, and the sensor’s resolution not being fine enough, which can result in errors.

However, the change in force distribution versus force ratio is consistent for the simulation, ANN model, and experiment. The result shows that by using backup roller with a suitable force ratio, the optimal non-uniformity can be reached. From there, the experimental results prove that the simulation results and the ANN model are reliable for prediction for a large-area R2R system with backup roller.

## 7. Discussion and Consequences

The simulation results show the influence of the geometric parameters on the force distribution in the conventional R2R system, of which the largest effect is the roller length with 84.87% contribution, followed by the roller diameter with 12.66%, and finally the thickness with the lowest contribution at 0.9%. Therefore, the rubber roller length should be short to improve the uniformity at the contact area. However, we cannot reduce the length of the roller because it depends on the target imprinting area. Moreover, the length of the roller should be as large as possible to increase the throughput and processing on the large-area pattern. Through simulation results and regression equation for the conventional R2R system, it can be seen that when the length is greater than 1200 mm, the uniformity becomes worse and it needs a method to compensate the bending. In this research, the backup roller was used to eliminate the bending deformation. Through the large-area R2R system with the support of backup roller simulation results, the force uniformity is significantly improved by using the backup roller with a suitable ratio. The optimal non-uniformity is lower than 3% for all lengths of rubber roller up to 2000 mm with the support of suitable backup roller length and optimal ratio. The simulation results also show that the force ratio R = 3 is the universal ratio, which means it can be used for all cases of rubber roller and backup roller to obtain good uniformity with a non-uniformity U less than 10%. 

Notably, ANN models were also used to train simulation data and make predictions not only on non-uniformity but also on the force distribution along the roller with small error. Most of the cases were predicted accurately in terms of bending direction, bending level, and vertical translation corresponding to the magnitude of the input force along with the geometry parameters of the R2R system. The experiment was also implemented to verify the simulation and ANN model; it proved the reliability of the ANN model for the prediction of a large-area R2R system with a backup roller. Therefore, this ANN model can then be used to predict any large-area R2R system in reality.

## 8. Conclusions

In summary, we conducted a detailed investigation on an R2R system, specifically, a large-scale R2R system. A series of simulations were carried out to investigate the influence of geometric parameters on the force distribution of the imprinting module. The results can be applied on any R2R system to predict the uniformity that the system can perform, thereby making judgments about processing ability to consider whether to use a backup roller to compensate the bending or not. In addition, the large-area R2R system with the support of the backup roller was also studied meticulously including the effect of imprinting roller length, backup roller length, average applied force, and force ratio on the force distribution. In particular, the ANN model was used to train the simulation data and had a good prediction of force distribution curve as well as non-uniformity in any cases within the survey range up to 2000 mm of imprinting area. From that, the model can provide an optimal recommendation on the selection of roller geometry as well as force ratio to eliminate the deformation in a specific large-area R2R system.

## Figures and Tables

**Table 1:** Geometry factors and levels.

Level	Rubber Roller Diameter D (mm)	Rubber Roller Thickness T (mm)	Rubber Roller Length L (mm)
1	130	9	800
2	160	12	1200
3	190	15	1600

**Table 2:** Grey relational grade and grey order.

No.	D	T	L	U (%)	Normalized Data (x_i_)	$\mathrm{Deviation}\;\mathrm{Sequence}\;({∆}_{i})$	Grey Relational Grade (GRG)	Grey Order
#1	1	1	1	4.6702	0.9403	0.0597	0.8934	4
#2	1	2	2	16.7003	0.6666	0.3334	0.6000	6
#3	1	3	3	45.996	0.0000	1.0000	0.3333	9
#4	2	1	2	11.1702	0.7924	0.2076	0.7066	5
#5	2	2	3	27.3834	0.4235	0.5765	0.4645	8
#6	2	3	1	2.0948	0.9989	0.0011	0.9979	2
#7	3	1	3	21.175	0.5648	0.4352	0.5346	7
#8	3	2	1	2.0479	1.0000	0.0000	1.0000	1
#9	3	3	2	4.6652	0.9404	0.0596	0.8936	3

**Table 3:** Respond table for grey relational grade.

	Level 1	Level 2	Level 3	Max–Min	Rank
D	0.6089	0.7230	**0.8094**	0.2005	2
T	0.7116	0.6881	**0.7416**	0.0534	3
L	**0.9638**	0.7334	0.4441	0.5196	1

**Table 4:** Results of ANOVA on grey relational grade.

	df	Sum of Squares	Mean Squares	F Value	Contribution
D	2	0.06069	0.030345	8.1	12.66%
T	2	0.004307	0.002154	0.57	0.90%
L	2	0.406718	0.203359	54.29	84.87%
Error	2	0.007491	0.003745		
Total	8	0.479206	0.239603		

**Table 5:** Factors and levels combination for simulation.

Factor	Levels	Values
Rubber roller length L (mm)	5	1200, 1400, 1600, 1800, 2000
Backup roller length B (mm)	5	200, 400, 600, 800, 1000
Force ratio R	5	1, 3, 5, 7, 9

**Table 6:** Summary of ANN model parameters for non-uniformity prediction.

Factor	Parameter
Model	Sequential model
Kernal initializer	He_uniform
Activation	Relu
Optimizer	Adam
Type of network	Feed forward back propagation
Loss function	Mean Squared Error
Batch size	15
Training rate	0.001
Number of epochs	1000
Number of input neurons	3
Number of hidden neurons	8
Number of hidden layers	2
Number of output neurons	1

**Table 7:** Factors and levels for simulation.

Factor	Levels	Values
Average applied force f (N/mm)	4	1, 3, 5, 7
Rubber roller length L (mm)	3	1200, 1600, 2000
Backup roller length B (mm)	3	200, 600, 1000
Force ratio R	3	1, 5, 9

## Data Availability

Not applicable.

## Supplementary Materials

The following supporting information can be downloaded at: https://www.mdpi.com/article/10.3390/polym15132909/s1; Supplementary S1 includes Figure S1: Stress concentration was shown on the cross section at the both ends of roller when using 20 mm axis diameter with average applied force f = 3 N/mm on the rubber roller length L = 1200 mm and D = 160 mm; Figure S2: Geometry parameters of rubber roller from the cross section view; Figure S3: Geometry parameters of backup roller from the cross section view; Figure S4: Training process of model with constant learning rate 0.025 (a) and learning rate 0.025 with exponential decay 0.99 (b); Figure S5: Comparison between prediction and target data of A (a) and C (b) for all cases with constant learning rate 0.025 model; Figure S6: Pressure mapping thin-film arrays sensor parameters; Table S1: Material properties; Table S2: Parameters of FEM models comparison; Table S3: Non-uniformity versus various axis diameter with average applied force f = 3 N/mm on the rubber roller length L = 1200 mm and D = 160 mm; Table S4: L9 orthogonal array with factor levels and results; Table S5: Non-uniformity simulation results of full factorial design of experiments; Table S6: Distributed curve results of full factorial design of experiment; Table S7: Summary of ANN model parameters for distributed curve prediction; and Appendix S1. Supplementary S2 presents a comparison plot between the predicted and target data, using the coefficient A and the intercept C as parameters. Supplementary S3 displays a comparison plot between the predicted and target data, where the coefficient A and residual error of intercept C are used as parameters.
