# Supplementary material for: Multi-Geometry Parameters Optimization of Large-Area Roll-to-Roll Nanoimprint Module Using Grey Relational Analysis and Artificial Neural Network
Source: Polymers (Basel). 2023 Jun 30;15(13):2909. doi: 10.3390/polym15132909 (PMC10346201; doi:10.3390/polym15132909)
Supplement: Supplementary file 1 [file polymers-15-02909-s001.zip › Supplementary_S1.pdf]

Table S1. Material properties

Material	Property	Value
Steel	Young's modulus (MPa)	200,000
	Poisson's ratio	0.3
Rubber (room-temperature-vulcanizing silicone)	Hardness (Shore A)	75
	Young's modulus (MPa)	7.05
	Poisson's ratio of rubber	0.49

Table S2. Parameters of FEM models comparison

Model	Nodes	Elements	Computing time (s)
Full model (Model a)	7,328,300	5,061,504	12600
Model with simplified master roller (Model b)	3,166,304	2,094,864	5580
Model with symmetric condition (Model c)	791,576	523,716	3694

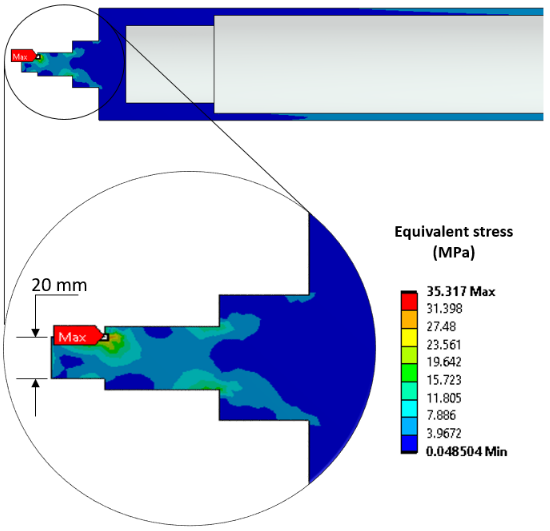

Figure S1. Stress concentration was shown on the cross section at the both ends of roller when using 20 mm axis diameter with average applied force f = 3 N/mm on the rubber roller length L = 1200 mm and D = 160 mm

Table S3. Non-uniformity versus various axis diameter with average applied force f = 3 N/mm on the rubber roller length L = 1200 mm and D = 160 mm

Axis diameter (mm)	Non-uniformity U (%)
20	11.5250
30	11.1702
40	10.6297
50	9.9125
60	9.1358

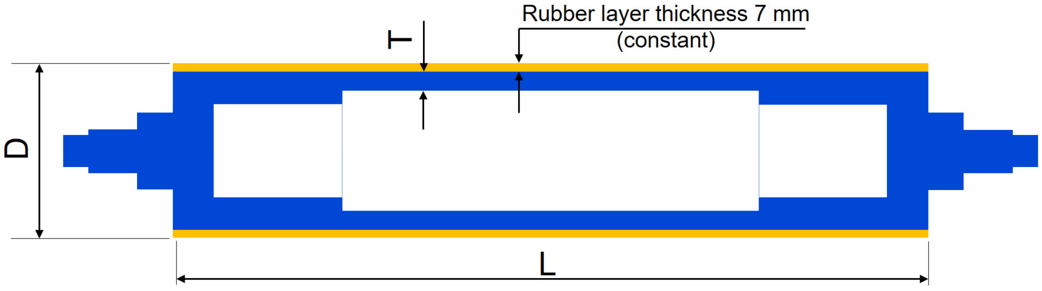

Figure S2. Geometry parameters of rubber roller from the cross section view

Table S4. L9 orthogonal array with factor levels and results.

No.	Rubber roller diameter D	Rubber roller thickness T	Rubber roller length L	Non-Uniformity U (%)
#1	1	1	1	4.6702
#2	1	2	2	16.7003
#3	1	3	3	45.996
#4	2	1	2	11.1702
#5	2	2	3	27.3834
#6	2	3	1	2.0948
#7	3	1	3	21.175
#8	3	2	1	2.0479
#9	3	3	2	4.6652

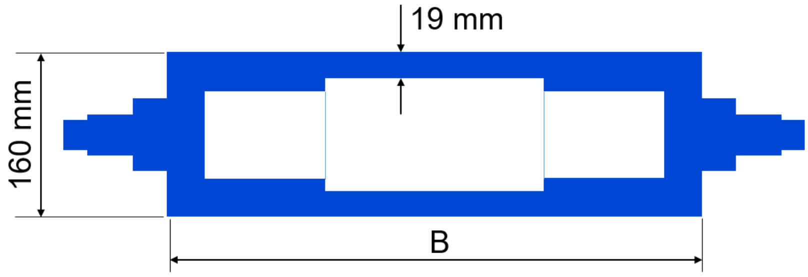

Figure S3. Geometry parameters of backup roller from the cross section view

Table S5. Non-uniformity simulation results of full factorial design of experiments

#	Rubber roller length L (mm)	Backup roller length B (mm)	Force ratio R	Non-uniformity U (%)
1	1200	200	1	3.243
2	1200	200	3	1.1567
3	1200	200	5	2.2205
4	1200	200	7	2.7811
5	1200	200	9	3.1158
6	1200	400	1	3.2806
7	1200	400	3	1.0237

#	Rubber roller length L (mm)	Backup roller length B (mm)	Force ratio R	Non-uniformity U (%)
8	1200	400	5	1.7829
9	1200	400	7	2.2811
10	1200	400	9	2.5813
11	1200	600	1	3.3897
12	1200	600	3	1.1485
13	1200	600	5	1.2262
14	1200	600	7	1.6837
15	1200	600	9	1.9554
16	1200	800	1	3.7794
17	1200	800	3	1.5031
18	1200	800	5	1.0039
19	1200	800	7	1.0741
20	1200	800	9	1.1909
21	1200	1000	1	4.2369
22	1200	1000	3	2.0865
23	1200	1000	5	1.4699
24	1200	1000	7	1.2231
25	1200	1000	9	1.1041
26	1400	200	1	5.386
27	1400	200	3	2.2132
28	1400	200	5	3.879
29	1400	200	7	4.9513
30	1400	200	9	5.5926
31	1400	400	1	5.3377
32	1400	400	3	1.9528
33	1400	400	5	3.1413
34	1400	400	7	4.1155
35	1400	400	9	4.7088
36	1400	600	1	5.55
37	1400	600	3	1.6143
38	1400	600	5	2.5096
39	1400	600	7	3.1767
40	1400	600	9	3.7114
41	1400	800	1	6.0419
42	1400	800	3	1.3464
43	1400	800	5	1.972
44	1400	800	7	2.3924
45	1400	800	9	2.6403
46	1400	1000	1	6.711
47	1400	1000	3	2.3205
48	1400	1000	5	1.337
49	1400	1000	7	1.7105
50	1400	1000	9	1.9327
51	1600	200	1	8.3297
52	1600	200	3	3.0437
53	1600	200	5	6.6049

#	Rubber roller length L (mm)	Backup roller length B (mm)	Force ratio R	Non-uniformity U (%)
54	1600	200	7	8.4514
55	1600	200	9	9.5907
56	1600	400	1	8.0975
57	1600	400	3	2.6938
58	1600	400	5	4.2506
59	1600	400	7	7.2452
60	1600	400	9	8.2466
61	1600	600	1	8.2817
62	1600	600	3	2.1526
63	1600	600	5	4.3782
64	1600	600	7	5.9203
65	1600	600	9	6.864
66	1600	800	1	8.8241
67	1600	800	3	2.7132
68	1600	800	5	3.0095
69	1600	800	7	4.3916
70	1600	800	9	5.2515
71	1600	1000	1	9.7198
72	1600	1000	3	3.5585
73	1600	1000	5	2.3486
74	1600	1000	7	2.7167
75	1600	1000	9	3.5244
76	1800	200	1	12.1742
77	1800	200	3	5.159
78	1800	200	5	10.7993
79	1800	200	7	13.6539
80	1800	200	9	15.3178
81	1800	400	1	11.8404
82	1800	400	3	4.2665
83	1800	400	5	9.4275
84	1800	400	7	12.0924
85	1800	400	9	13.6764
86	1800	600	1	11.9996
87	1800	600	3	3.1865
88	1800	600	5	7.8623
89	1800	600	7	10.3419
90	1800	600	9	11.8439
91	1800	800	1	12.4608
92	1800	800	3	2.1355
93	1800	800	5	6.0213
94	1800	800	7	8.3207
95	1800	800	9	9.7178
96	1800	1000	1	13.6866
97	1800	1000	3	1.5054
98	1800	1000	5	4.016
99	1800	1000	7	6.0297

#	Rubber roller length L (mm)	Backup roller length B (mm)	Force ratio R	Non-uniformity U (%)
100	1800	1000	9	7.3281
101	2000	200	1	18.0742
102	2000	200	3	7.7704
103	2000	200	5	15.8905
104	2000	200	7	19.825
105	2000	200	9	22.1766
106	2000	400	1	17.4394
107	2000	400	3	6.5714
108	2000	400	5	14.1571
109	2000	400	7	18.0047
110	2000	400	9	20.1933
111	2000	600	1	17.519
112	2000	600	3	5.1093
113	2000	600	5	12.1712
114	2000	600	7	15.8748
115	2000	600	9	18.0626
116	2000	800	1	18.0836
117	2000	800	3	3.7569
118	2000	800	5	9.8848
119	2000	800	7	13.3307
120	2000	800	9	15.4368
121	2000	1000	1	19.3908
122	2000	1000	3	2.1807
123	2000	1000	5	7.3055
124	2000	1000	7	10.4359
125	2000	1000	9	12.4108

Table S6. Distributed curve results of full factorial design of experiment

#	Average applied force f (N/mm)	Rubber roller length L (mm)	Backup roller length B (mm)	Force ratio R	A (x10^{-7})	C
1	1	1200	200	1	0.7310	1.0042
2	1	1200	200	5	-0.3430	1.0228
3	1	1200	200	9	-0.5680	1.0266
4	1	1200	600	1	0.8590	1.0018
5	1	1200	600	5	-0.1140	1.0181
6	1	1200	600	9	-0.3080	1.0213
7	1	1200	1000	1	1.0880	0.9979
8	1	1200	1000	5	0.2850	1.0114
9	1	1200	1000	9	0.1210	1.0142
10	1	1600	200	1	1.0190	0.9869
11	1	1600	200	5	-1.0160	1.0426
12	1	1600	200	9	-1.4520	1.0544
13	1	1600	600	1	1.1360	0.9833
14	1	1600	600	5	-0.6820	1.0327
15	1	1600	600	9	-1.0430	1.0425

#	Average applied force f (N/mm)	Rubber roller length L (mm)	Backup roller length B (mm)	Force ratio R	A ($\times 10^{-7}$)	C
16	1	1600	1000	1	1.4120	0.9758
17	1	1600	1000	5	-0.2160	1.0204
18	1	1600	1000	9	-0.5440	1.0292
19	1	2000	200	1	1.4160	0.9562
20	1	2000	200	5	-1.7330	1.0831
21	1	2000	200	9	-2.3890	1.1093
22	1	2000	600	1	1.4750	0.9533
23	1	2000	600	5	-1.2950	1.0652
24	1	2000	600	9	-1.8720	1.0881
25	1	2000	1000	1	1.7590	0.9420
26	1	2000	1000	5	-0.8090	1.0459
27	1	2000	1000	9	-1.3200	1.0663
28	3	1200	200	1	3.6750	2.9683
29	3	1200	200	5	-1.9800	3.0643
30	3	1200	200	9	-3.1880	3.0847
31	3	1200	600	1	3.8780	2.9614
32	3	1200	600	5	-0.7230	3.0401
33	3	1200	600	9	-1.6950	3.0565
34	3	1200	1000	1	5.0020	2.9428
35	3	1200	1000	5	1.1760	3.0069
36	3	1200	1000	9	0.4520	3.0197
37	3	1600	200	1	5.3420	2.8796
38	3	1600	200	5	-5.3790	3.1716
39	3	1600	200	9	-7.6650	3.2336
40	3	1600	600	1	5.4580	2.8742
41	3	1600	600	5	-3.4880	3.1194
42	3	1600	600	9	-5.4280	3.1721
43	3	1600	1000	1	6.6180	2.8427
44	3	1600	1000	5	-1.0520	3.0530
45	3	1600	1000	9	-2.6530	3.0967
46	3	2000	200	1	7.1730	2.7303
47	3	2000	200	5	-8.8870	3.3777
48	3	2000	200	9	-12.2980	3.5132
49	3	2000	600	1	7.1420	2.7312
50	3	2000	600	5	-6.7190	3.2920
51	3	2000	600	9	-9.7290	3.4124
52	3	2000	1000	1	8.2060	2.6889
53	3	2000	1000	5	-3.9330	3.1815
54	3	2000	1000	9	-6.5140	3.2856
55	5	1200	200	1	8.6700	4.9442
56	5	1200	200	5	-4.2100	5.1582
57	5	1200	200	9	-6.8500	5.2018
58	5	1200	600	1	8.9600	4.9322
59	5	1200	600	5	-1.5500	5.1116
60	5	1200	600	9	-3.8400	5.1504

#	Average applied force f (N/mm)	Rubber roller length L (mm)	Backup roller length B (mm)	Force ratio R	A $(x10^{-7})$	C
61	5	1200	1000	1	11.4000	4.8910
62	5	1200	1000	5	2.8600	5.0346
63	5	1200	1000	9	1.1500	5.0641
64	5	1600	200	1	12.0000	4.7488
65	5	1600	200	5	-11.6000	5.3899
66	5	1600	200	9	-16.7000	5.5247
67	5	1600	600	1	12.3000	4.7378
68	5	1600	600	5	-7.9900	5.2926
69	5	1600	600	9	-12.3000	5.4093
70	5	1600	1000	1	15.0000	4.6662
71	5	1600	1000	5	-2.3300	5.1391
72	5	1600	1000	9	-5.9600	5.2385
73	5	2000	200	1	15.4000	4.4410
74	5	2000	200	5	-19.7000	5.8530
75	5	2000	200	9	-27.2000	6.1517
76	5	2000	600	1	15.5000	4.4399
77	5	2000	600	5	-14.6000	5.6526
78	5	2000	600	9	-20.3000	5.8798
79	5	2000	1000	1	17.8000	4.3444
80	5	2000	1000	5	-8.5900	5.4182
81	5	2000	1000	9	-14.1000	5.6396
82	7	1200	200	1	13.6790	6.8771
83	7	1200	200	5	-7.1940	7.2363
84	7	1200	200	9	-11.7720	7.3153
85	7	1200	600	1	14.9750	6.8452
86	7	1200	600	5	-2.4580	7.1470
87	7	1200	600	9	-6.2390	7.2113
88	7	1200	1000	1	19.1450	6.7763
89	7	1200	1000	5	4.8540	7.0148
90	7	1200	1000	9	2.0370	7.0639
91	7	1600	200	1	19.8640	6.5560
92	7	1600	200	5	-19.2900	7.6169
93	7	1600	200	9	-27.5670	7.8400
94	7	1600	600	1	20.4150	6.5297
95	7	1600	600	5	-13.0850	7.4474
96	7	1600	600	9	-19.7340	7.6238
97	7	1600	1000	1	24.8140	6.4098
98	7	1600	1000	5	-3.6900	7.1891
99	7	1600	1000	9	-9.6450	7.3533
100	7	2000	200	1	25.3890	6.0551
101	7	2000	200	5	-30.4990	8.3011
102	7	2000	200	9	-42.2760	8.7637
103	7	2000	600	1	25.0740	6.0597
104	7	2000	600	5	-22.9510	7.9991
105	7	2000	600	9	-32.3430	8.3695

#	Average applied force f (N/mm)	Rubber roller length L (mm)	Backup roller length B (mm)	Force ratio R	A (x10^{-7})	C
106	7	2000	1000	1	29.1650	5.8947
107	7	2000	1000	5	-13.8070	7.6412
108	7	2000	1000	9	-22.6470	7.9663

Table S7. Summary of ANN model parameters for distributed curve prediction

Factor	Parameter
Model	Sequential model
Kernal initializer	He_uniform
Activation	Relu
Optimizer	Adam
Type of network	Feed forward back propagation
Loss function	Mean Squared Error
Batch size	15
Training rate	0.025 (constant)<br>0.025 (0.99 decay rate)
Number of epochs	1000
Training data	81
Validation data	27
Number of input neurons	4
Number of hidden neurons	16, 32, 16
Number of hidden layer	3
Number of output neurons	2

The parameters of ANN model used for distributed curve along the roller via coefficient A, C are described in Table S7. The dataset comprises 108 cases, which are divided into 75% training data (81 cases) and 25% testing data (27 cases) using radom_state = 42 this ensures that the dataset will be split in the same way every time the code is executed. The input features are scaled using the standard scaler from the sklearn preprocessing library. Additionally, the coefficient A of the curve equation is scaled by multiplying it by 10^7 because the magnitude of the data is too small, which can potentially cause issues in training and prediction accuracy. The scaler inverse transform will be used in the prediction step to return the values to the original scale. After examining many different numbers, the 15 batch sizes and 3 hidden layers were chosen with 16, 32, and 16 neurons for each hidden layer, respectively. Choosing an appropriate learning rate is also crucial in training ANNs, as an inappropriate learning rate can impact the convergence speed, training stability, and the quality of the trained model and it is set at 0.025 in this model

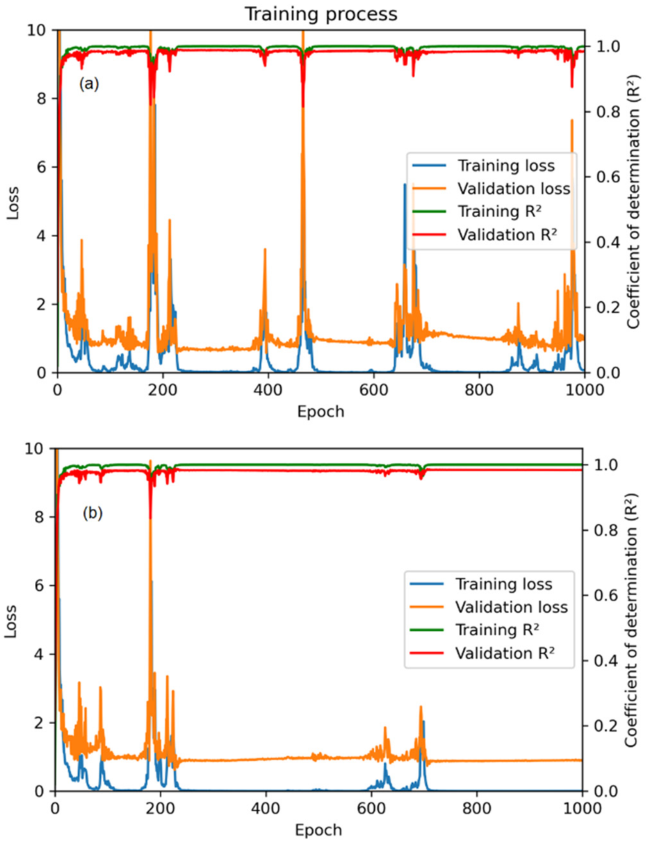

Figure S4. Training process of model with constant learning rate 0.025 (a) and learning rate 0.025 with exponential decay 0.99 (b)

Loss and coefficient of determination were used as the performance measurement of the two models. By using learning rate with exponential decay the model performs better with more stable in loss function during training process. The same goes for coefficient of determination (R2), both models have an R2 value close to 1 with a smoother curve when using learning rate with decay. The results indicate that the model can capture the variance in the target variable and has a good fit for the dataset.

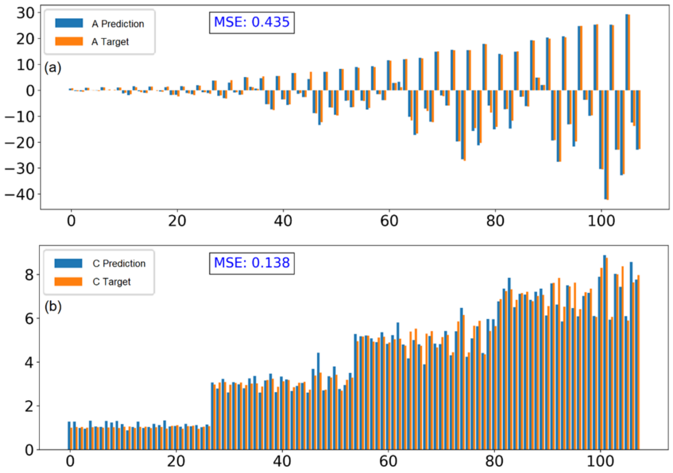

Figure S5. Comparison between prediction and target data of A (a) and C (b) for all cases with constant learning rate 0.025 model

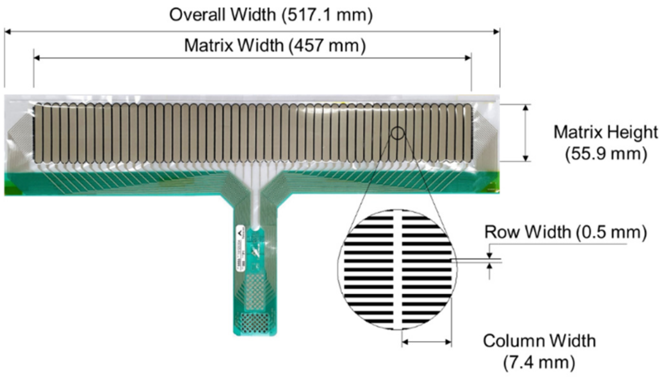

Figure S6. Pressure mapping thin-film arrays sensor parameters

Appendix S1

The analysis of variance (ANOVA) for the Taguchi design of data in table 2 for GRG. The table can be obtained by calculated the **sum of square (SS)** of each parameter between groups

$$SS_{each\ group} = \sum n_i(x_i\text{-}\bar{x})^2$$

With n_i is the number of value in i^{th} group

x_i is the mean of the ith group

$\bar{x}$ is the overall mean

Mean square

$$MS = \frac{SS}{df}$$

with df = number of group – 1

Sum of square total (SS_{total}) includes : sum of square deviations due to each geometry parameters and sum of square error (SS_{error} or error).

$$SS_{total} = \sum (x\text{-}\bar{x})^2$$

With $\bar{x}$ is the overal mean, x is all individual observed value

Sum of square error (SS_{error} or error)

$$SS_{error}\ (error) = SS_{total}\text{-}\sum SS_{each\ group}$$

R-squared

$$R^2 = 1\text{-}\frac{SS_{error}}{SS_{total}}$$

The contribution of each geometry parameter can be calculated by sum of square of each parameter in the total sum of square deviation

The ANOVA table can be simply obtained by statistical software. (In this study we obtained by Minitab software)
