# Supplementary material for: Multi-Geometry Parameters Optimization of Large-Area Roll-to-Roll Nanoimprint Module Using Grey Relational Analysis and Artificial Neural Network
Source: Polymers (Basel). 2023 Jun 30;15(13):2909. doi: 10.3390/polym15132909 (PMC10346201; doi:10.3390/polym15132909)
Supplement: Supplementary file 1 [file polymers-15-02909-s001.zip › Supplementary_S3.pdf]

Comparison plot between the prediction and target data based on the the model trained with coefficient A and the residual error of C and the average applied force

Similarly to Supplementary 2. A series of graph below plot entire dataset of 108 cases, including 81 training cases (Train) and 27 validation cases (Test) using the model with learning rate of 0.025 and 0.99 exponential decay. However, the output was used in this case is the residual error between C and the average applied force instead of C as in previous model (model used in supplementary 2). The x-axis is represents the axial position of the roller, and the y-axis represents the distributed force f_x. For easier observation, all prediction equations (Prediction) and target equations (Target) are plot on the same axial range from -1000 to 1000. As the results shown in Figure 13, the prediction and target data of C are consistent. Therefore, most of cases were plotted below shown a very small discrepancy of vertical translation prove that this model improved the vertical translation error of force distribution curve prediction.

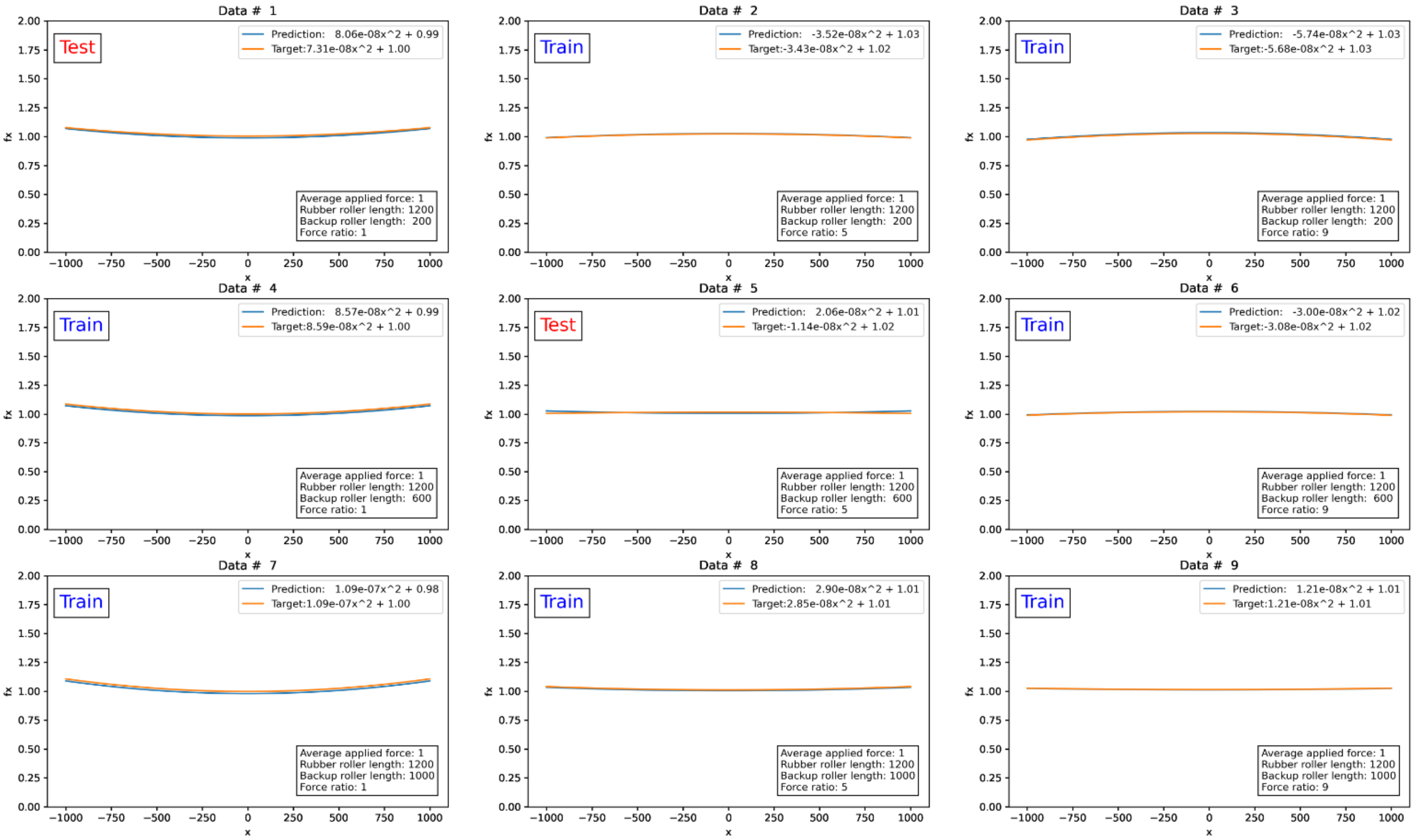
Data # 1
Test
Prediction: 8.06e-08x^2 + 0.99
Target:7.31e-08x^2 + 1.00
Average applied force: 1
Rubber roller length: 1200
Backup roller length: 200
Force ratio: 1
Data # 2
Train
Prediction: -3.52e-08x^2 + 1.03
Target:-3.43e-08x^2 + 1.02
Average applied force: 1
Rubber roller length: 1200
Backup roller length: 200
Force ratio: 5
Data # 3
Train
Prediction: -5.74e-08x^2 + 1.03
Target:-5.68e-08x^2 + 1.03
Average applied force: 1
Rubber roller length: 1200
Backup roller length: 200
Force ratio: 9
Data # 4
Train
Prediction: 8.57e-08x^2 + 0.99
Target:8.59e-08x^2 + 1.00
Average applied force: 1
Rubber roller length: 1200
Backup roller length: 600
Force ratio: 1
Data # 5
Test
Prediction: 2.06e-08x^2 + 1.01
Target:-1.14e-08x^2 + 1.02
Average applied force: 1
Rubber roller length: 1200
Backup roller length: 600
Force ratio: 5
Data # 6
Train
Prediction: -3.00e-08x^2 + 1.02
Target:-3.08e-08x^2 + 1.02
Average applied force: 1
Rubber roller length: 1200
Backup roller length: 600
Force ratio: 9
Data # 7
Train
Prediction: 1.09e-07x^2 + 0.98
Target:1.09e-07x^2 + 1.00
Average applied force: 1
Rubber roller length: 1200
Backup roller length: 1000
Force ratio: 1
Data # 8
Train
Prediction: 2.90e-08x^2 + 1.01
Target:2.85e-08x^2 + 1.01
Average applied force: 1
Rubber roller length: 1200
Backup roller length: 1000
Force ratio: 5
Data # 9
Train
Prediction: 1.21e-08x^2 + 1.01
Target:1.21e-08x^2 + 1.01
Average applied force: 1
Rubber roller length: 1200
Backup roller length: 1000
Force ratio: 9
fx
x

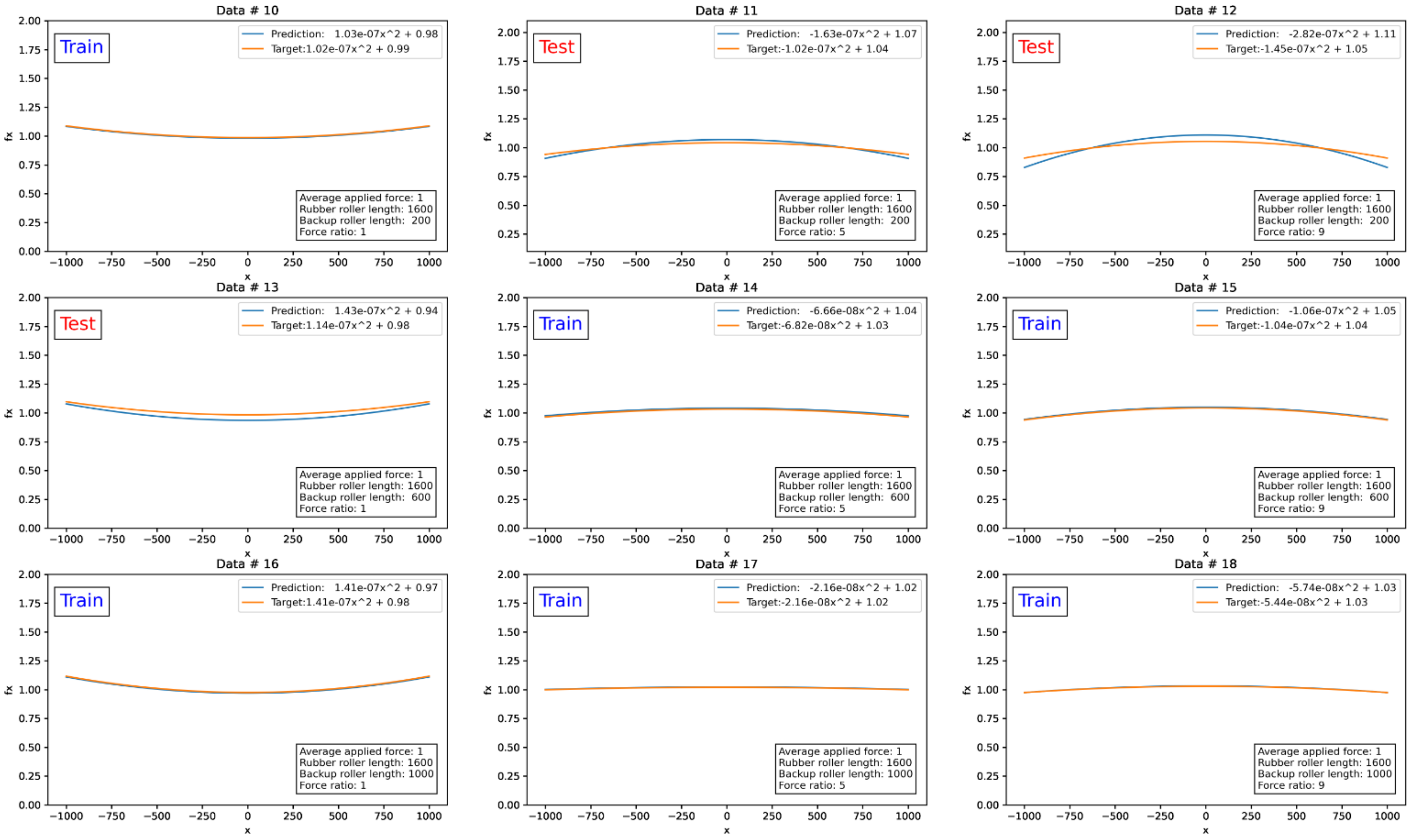
Data # 10
Train
Prediction: 1.03e-07x^2 + 0.98
Target:1.02e-07x^2 + 0.99
Average applied force: 1
Rubber roller length: 1600
Backup roller length: 200
Force ratio: 1
Data # 11
Test
Prediction: -1.63e-07x^2 + 1.07
Target:-1.02e-07x^2 + 1.04
Average applied force: 1
Rubber roller length: 1600
Backup roller length: 200
Force ratio: 5
Data # 12
Test
Prediction: -2.82e-07x^2 + 1.11
Target:-1.45e-07x^2 + 1.05
Average applied force: 1
Rubber roller length: 1600
Backup roller length: 200
Force ratio: 9
Data # 13
Test
Prediction: 1.43e-07x^2 + 0.94
Target:1.14e-07x^2 + 0.98
Average applied force: 1
Rubber roller length: 1600
Backup roller length: 600
Force ratio: 1
Data # 14
Train
Prediction: -6.66e-08x^2 + 1.04
Target:-6.82e-08x^2 + 1.03
Average applied force: 1
Rubber roller length: 1600
Backup roller length: 600
Force ratio: 5
Data # 15
Train
Prediction: -1.06e-07x^2 + 1.05
Target:-1.04e-07x^2 + 1.04
Average applied force: 1
Rubber roller length: 1600
Backup roller length: 600
Force ratio: 9
Data # 16
Train
Prediction: 1.41e-07x^2 + 0.97
Target:1.41e-07x^2 + 0.98
Average applied force: 1
Rubber roller length: 1600
Backup roller length: 1000
Force ratio: 1
Data # 17
Train
Prediction: -2.16e-08x^2 + 1.02
Target:-2.16e-08x^2 + 1.02
Average applied force: 1
Rubber roller length: 1600
Backup roller length: 1000
Force ratio: 5
Data # 18
Train
Prediction: -5.74e-08x^2 + 1.03
Target:-5.44e-08x^2 + 1.03
Average applied force: 1
Rubber roller length: 1600
Backup roller length: 1000
Force ratio: 9
fx
x

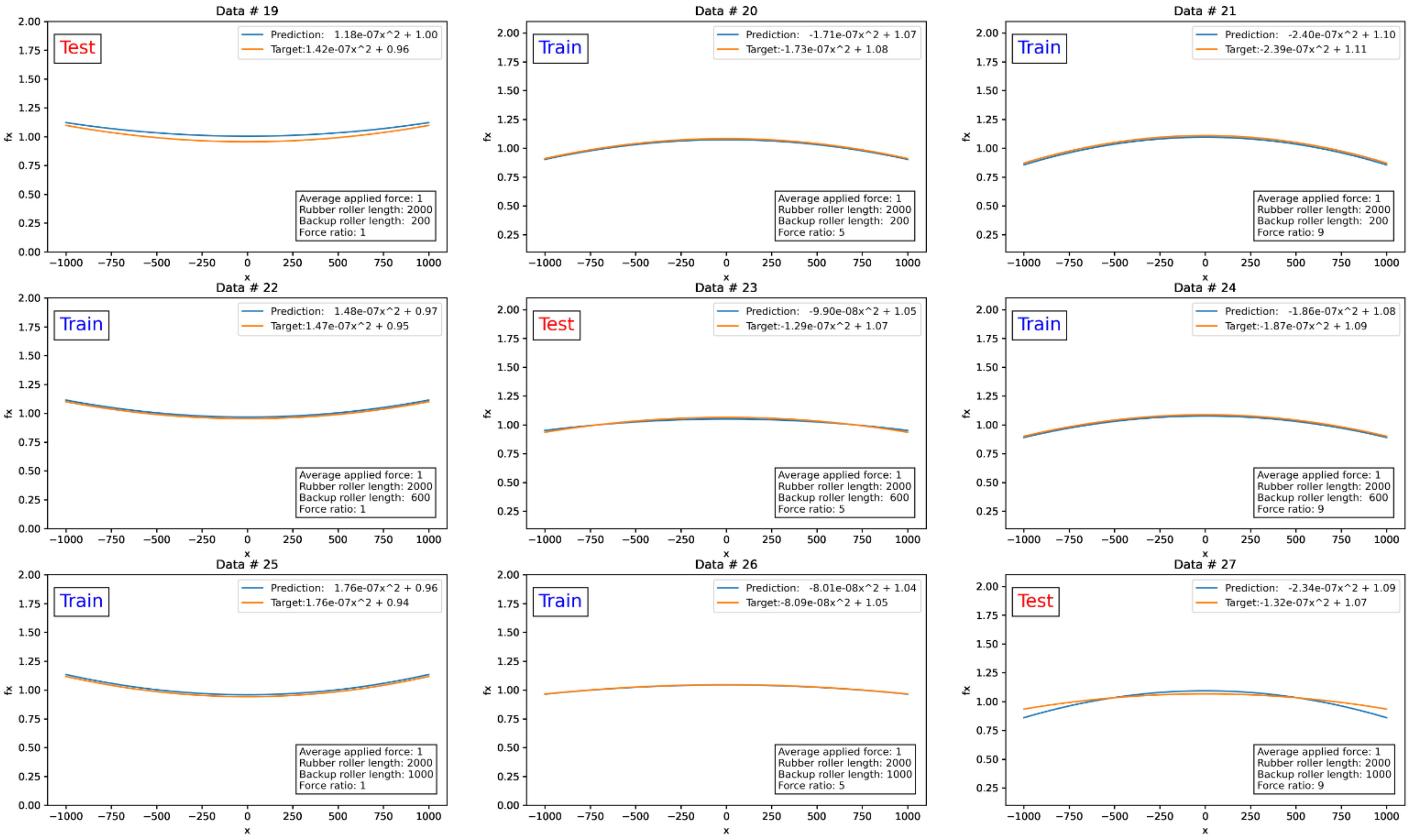
Data # 19
Test
Prediction: 1.18e-07x^2 + 1.00
Target:1.42e-07x^2 + 0.96
Average applied force: 1
Rubber roller length: 2000
Backup roller length: 200
Force ratio: 1
Data # 20
Train
Prediction: -1.71e-07x^2 + 1.07
Target:-1.73e-07x^2 + 1.08
Average applied force: 1
Rubber roller length: 2000
Backup roller length: 200
Force ratio: 5
Data # 21
Train
Prediction: -2.40e-07x^2 + 1.10
Target:-2.39e-07x^2 + 1.11
Average applied force: 1
Rubber roller length: 2000
Backup roller length: 200
Force ratio: 9
Data # 22
Train
Prediction: 1.48e-07x^2 + 0.97
Target:1.47e-07x^2 + 0.95
Average applied force: 1
Rubber roller length: 2000
Backup roller length: 600
Force ratio: 1
Data # 23
Test
Prediction: -9.90e-08x^2 + 1.05
Target:-1.29e-07x^2 + 1.07
Average applied force: 1
Rubber roller length: 2000
Backup roller length: 600
Force ratio: 5
Data # 24
Train
Prediction: -1.86e-07x^2 + 1.08
Target:-1.87e-07x^2 + 1.09
Average applied force: 1
Rubber roller length: 2000
Backup roller length: 600
Force ratio: 9
Data # 25
Train
Prediction: 1.76e-07x^2 + 0.96
Target:1.76e-07x^2 + 0.94
Average applied force: 1
Rubber roller length: 2000
Backup roller length: 1000
Force ratio: 1
Data # 26
Train
Prediction: -8.01e-08x^2 + 1.04
Target:-8.09e-08x^2 + 1.05
Average applied force: 1
Rubber roller length: 2000
Backup roller length: 1000
Force ratio: 5
Data # 27
Test
Prediction: -2.34e-07x^2 + 1.09
Target:-1.32e-07x^2 + 1.07
Average applied force: 1
Rubber roller length: 2000
Backup roller length: 1000
Force ratio: 9
fx
x

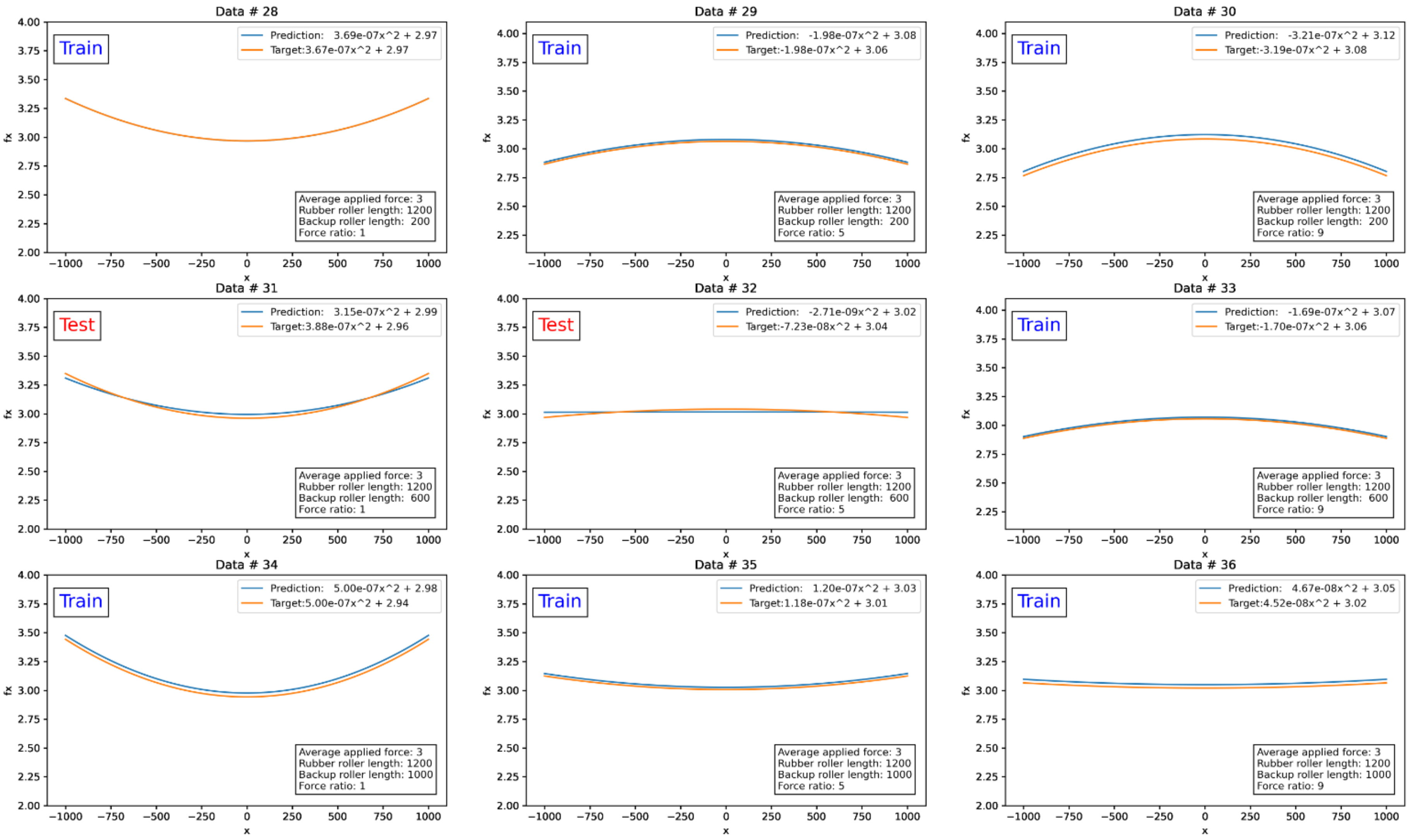

Data # 28
Train
Prediction: 3.69e-07x^2 + 2.97
Target:3.67e-07x^2 + 2.97
Average applied force: 3
Rubber roller length: 1200
Backup roller length: 200
Force ratio: 1
Data # 29
Train
Prediction: -1.98e-07x^2 + 3.08
Target:-1.98e-07x^2 + 3.06
Average applied force: 3
Rubber roller length: 1200
Backup roller length: 200
Force ratio: 5
Data # 30
Train
Prediction: -3.21e-07x^2 + 3.12
Target:-3.19e-07x^2 + 3.08
Average applied force: 3
Rubber roller length: 1200
Backup roller length: 200
Force ratio: 9
Data # 31
Test
Prediction: 3.15e-07x^2 + 2.99
Target:3.88e-07x^2 + 2.96
Average applied force: 3
Rubber roller length: 1200
Backup roller length: 600
Force ratio: 1
Data # 32
Test
Prediction: -2.71e-09x^2 + 3.02
Target:-7.23e-08x^2 + 3.04
Average applied force: 3
Rubber roller length: 1200
Backup roller length: 600
Force ratio: 5
Data # 33
Train
Prediction: -1.69e-07x^2 + 3.07
Target:-1.70e-07x^2 + 3.06
Average applied force: 3
Rubber roller length: 1200
Backup roller length: 600
Force ratio: 9
Data # 34
Train
Prediction: 5.00e-07x^2 + 2.98
Target:5.00e-07x^2 + 2.94
Average applied force: 3
Rubber roller length: 1200
Backup roller length: 1000
Force ratio: 1
Data # 35
Train
Prediction: 1.20e-07x^2 + 3.03
Target:1.18e-07x^2 + 3.01
Average applied force: 3
Rubber roller length: 1200
Backup roller length: 1000
Force ratio: 5
Data # 36
Train
Prediction: 4.67e-08x^2 + 3.05
Target:4.52e-08x^2 + 3.02
Average applied force: 3
Rubber roller length: 1200
Backup roller length: 1000
Force ratio: 9
fx
x

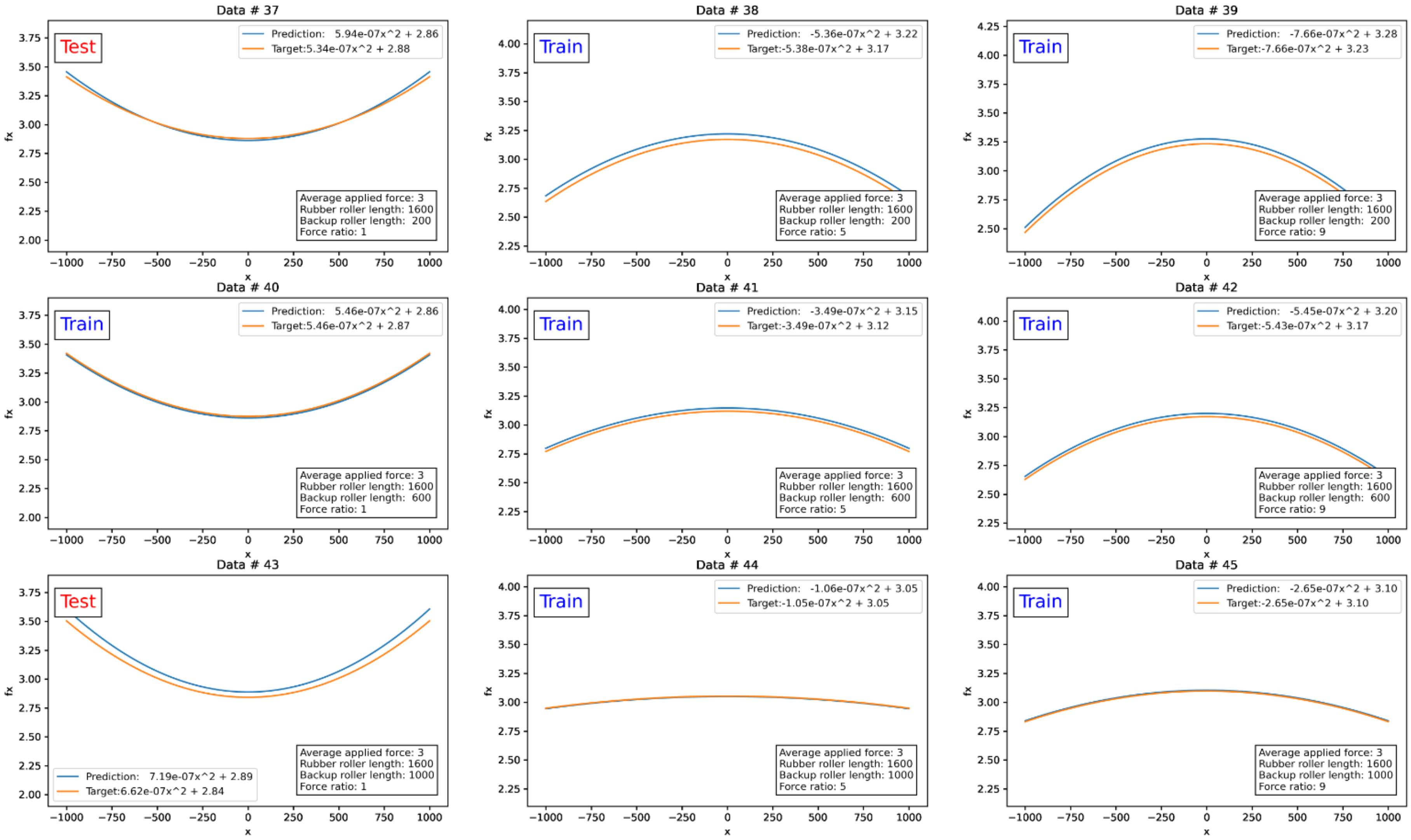
Data # 37
Test
Prediction: 5.94e-07x^2 + 2.86
Target:5.34e-07x^2 + 2.88
Average applied force: 3
Rubber roller length: 1600
Backup roller length: 200
Force ratio: 1
Data # 38
Train
Prediction: -5.36e-07x^2 + 3.22
Target:-5.38e-07x^2 + 3.17
Average applied force: 3
Rubber roller length: 1600
Backup roller length: 200
Force ratio: 5
Data # 39
Train
Prediction: -7.66e-07x^2 + 3.28
Target:-7.66e-07x^2 + 3.23
Average applied force: 3
Rubber roller length: 1600
Backup roller length: 200
Force ratio: 9
Data # 40
Train
Prediction: 5.46e-07x^2 + 2.86
Target:5.46e-07x^2 + 2.87
Average applied force: 3
Rubber roller length: 1600
Backup roller length: 600
Force ratio: 1
Data # 41
Train
Prediction: -3.49e-07x^2 + 3.15
Target:-3.49e-07x^2 + 3.12
Average applied force: 3
Rubber roller length: 1600
Backup roller length: 600
Force ratio: 5
Data # 42
Train
Prediction: -5.45e-07x^2 + 3.20
Target:-5.43e-07x^2 + 3.17
Average applied force: 3
Rubber roller length: 1600
Backup roller length: 600
Force ratio: 9
Data # 43
Test
Prediction: 7.19e-07x^2 + 2.89
Target:6.62e-07x^2 + 2.84
Average applied force: 3
Rubber roller length: 1600
Backup roller length: 1000
Force ratio: 1
Data # 44
Train
Prediction: -1.06e-07x^2 + 3.05
Target:-1.05e-07x^2 + 3.05
Average applied force: 3
Rubber roller length: 1600
Backup roller length: 1000
Force ratio: 5
Data # 45
Train
Prediction: -2.65e-07x^2 + 3.10
Target:-2.65e-07x^2 + 3.10
Average applied force: 3
Rubber roller length: 1600
Backup roller length: 1000
Force ratio: 9
fx
x

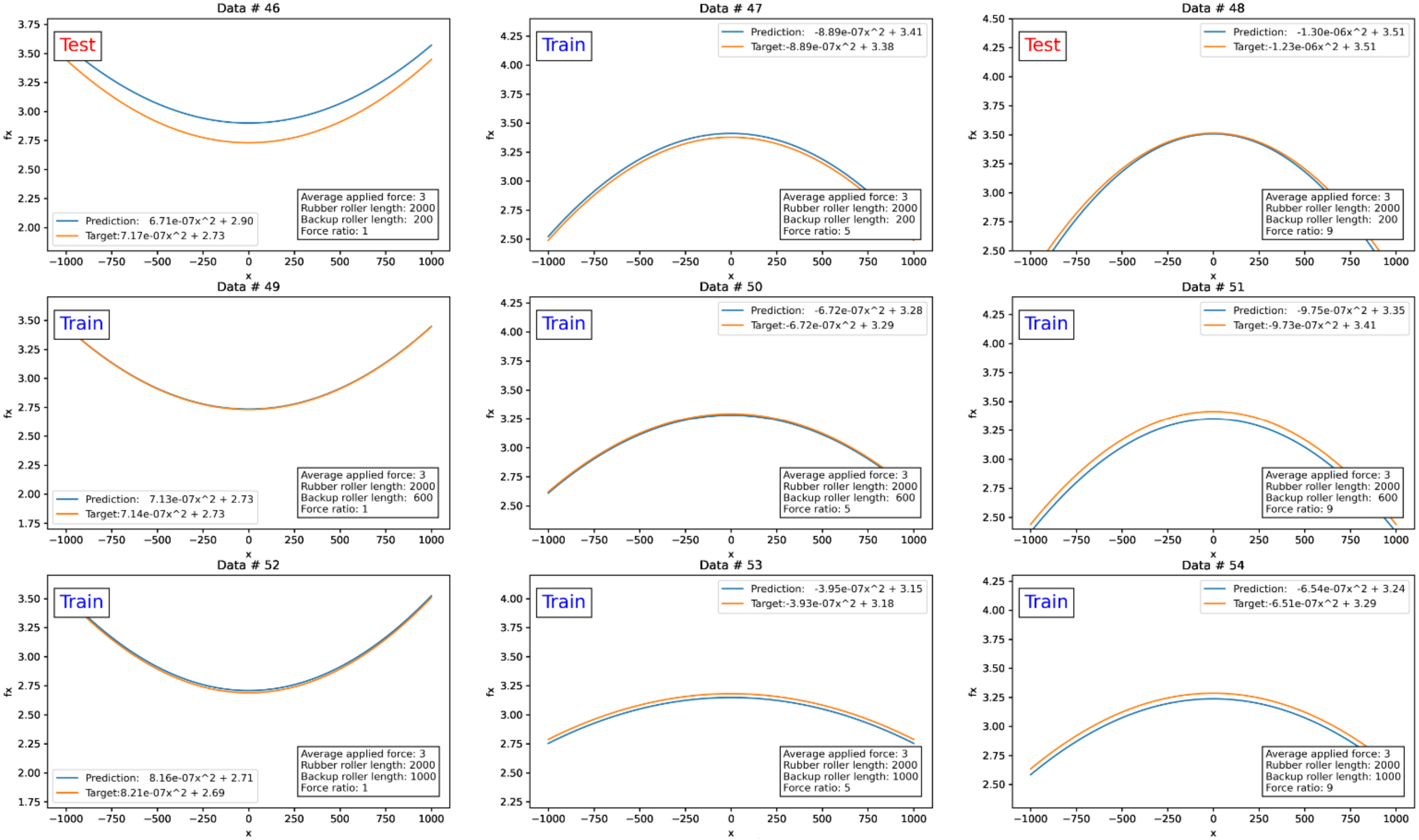
Data # 46
Test
Prediction: 6.71e-07x^2 + 2.90
Target:7.17e-07x^2 + 2.73
Average applied force: 3
Rubber roller length: 2000
Backup roller length: 200
Force ratio: 1
Data # 47
Train
Prediction: -8.89e-07x^2 + 3.41
Target:-8.89e-07x^2 + 3.38
Average applied force: 3
Rubber roller length: 2000
Backup roller length: 200
Force ratio: 5
Data # 48
Test
Prediction: -1.30e-06x^2 + 3.51
Target:-1.23e-06x^2 + 3.51
Average applied force: 3
Rubber roller length: 2000
Backup roller length: 200
Force ratio: 9
Data # 49
Train
Prediction: 7.13e-07x^2 + 2.73
Target:7.14e-07x^2 + 2.73
Average applied force: 3
Rubber roller length: 2000
Backup roller length: 600
Force ratio: 1
Data # 50
Train
Prediction: -6.72e-07x^2 + 3.28
Target:-6.72e-07x^2 + 3.29
Average applied force: 3
Rubber roller length: 2000
Backup roller length: 600
Force ratio: 5
Data # 51
Train
Prediction: -9.75e-07x^2 + 3.35
Target:-9.73e-07x^2 + 3.41
Average applied force: 3
Rubber roller length: 2000
Backup roller length: 600
Force ratio: 9
Data # 52
Train
Prediction: 8.16e-07x^2 + 2.71
Target:8.21e-07x^2 + 2.69
Average applied force: 3
Rubber roller length: 2000
Backup roller length: 1000
Force ratio: 1
Data # 53
Train
Prediction: -3.95e-07x^2 + 3.15
Target:-3.93e-07x^2 + 3.18
Average applied force: 3
Rubber roller length: 2000
Backup roller length: 1000
Force ratio: 5
Data # 54
Train
Prediction: -6.54e-07x^2 + 3.24
Target:-6.51e-07x^2 + 3.29
Average applied force: 3
Rubber roller length: 2000
Backup roller length: 1000
Force ratio: 9
fx
x

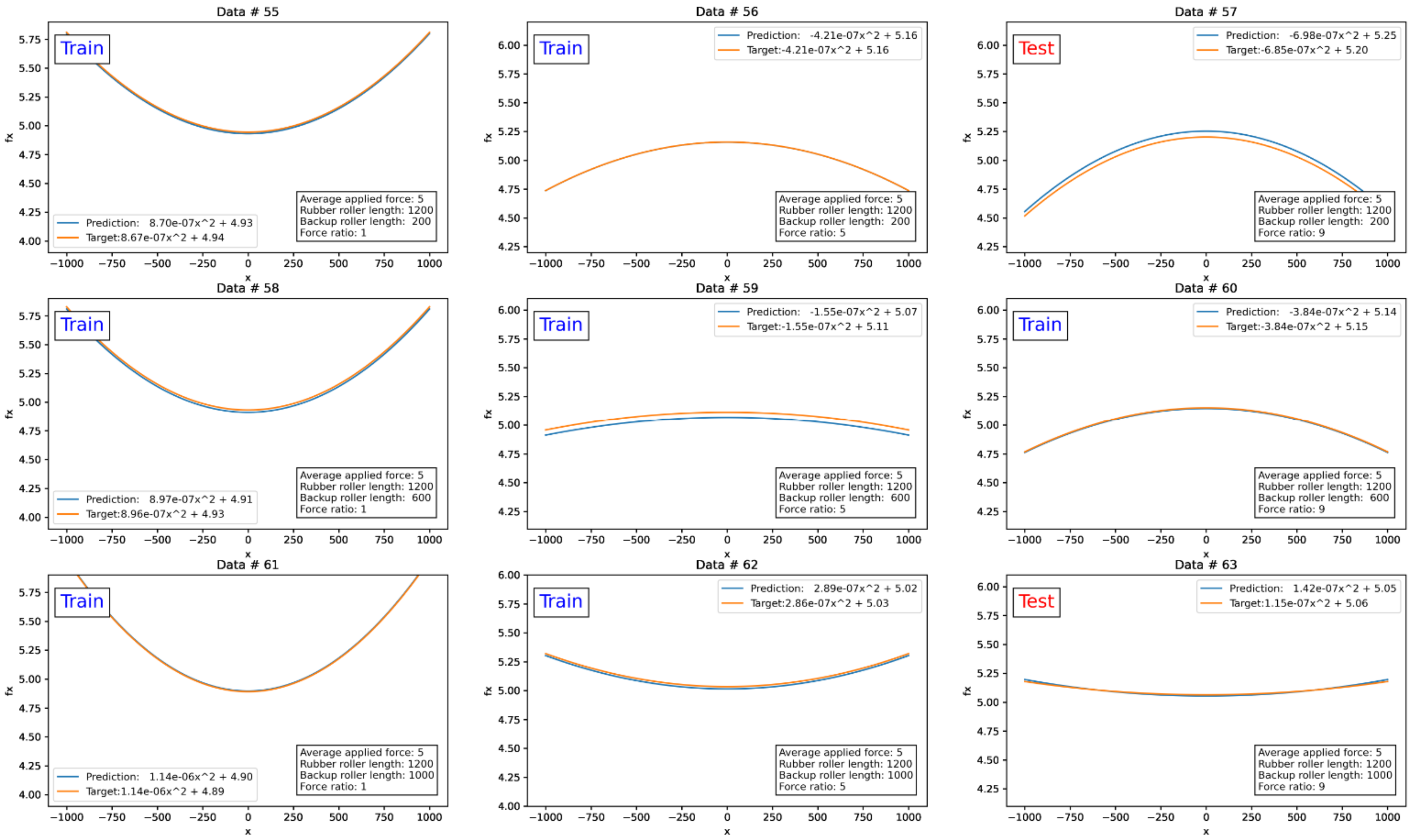
Data # 55
Train
Prediction: 8.70e-07x^2 + 4.93
Target:8.67e-07x^2 + 4.94
Average applied force: 5
Rubber roller length: 1200
Backup roller length: 200
Force ratio: 1
Data # 56
Train
Prediction: -4.21e-07x^2 + 5.16
Target:-4.21e-07x^2 + 5.16
Average applied force: 5
Rubber roller length: 1200
Backup roller length: 200
Force ratio: 5
Data # 57
Test
Prediction: -6.98e-07x^2 + 5.25
Target:-6.85e-07x^2 + 5.20
Average applied force: 5
Rubber roller length: 1200
Backup roller length: 200
Force ratio: 9
Data # 58
Train
Prediction: 8.97e-07x^2 + 4.91
Target:8.96e-07x^2 + 4.93
Average applied force: 5
Rubber roller length: 1200
Backup roller length: 600
Force ratio: 1
Data # 59
Train
Prediction: -1.55e-07x^2 + 5.07
Target:-1.55e-07x^2 + 5.11
Average applied force: 5
Rubber roller length: 1200
Backup roller length: 600
Force ratio: 5
Data # 60
Train
Prediction: -3.84e-07x^2 + 5.14
Target:-3.84e-07x^2 + 5.15
Average applied force: 5
Rubber roller length: 1200
Backup roller length: 600
Force ratio: 9
Data # 61
Train
Prediction: 1.14e-06x^2 + 4.90
Target:1.14e-06x^2 + 4.89
Average applied force: 5
Rubber roller length: 1200
Backup roller length: 1000
Force ratio: 1
Data # 62
Train
Prediction: 2.89e-07x^2 + 5.02
Target:2.86e-07x^2 + 5.03
Average applied force: 5
Rubber roller length: 1200
Backup roller length: 1000
Force ratio: 5
Data # 63
Test
Prediction: 1.42e-07x^2 + 5.05
Target:1.15e-07x^2 + 5.06
Average applied force: 5
Rubber roller length: 1200
Backup roller length: 1000
Force ratio: 9
fx
x

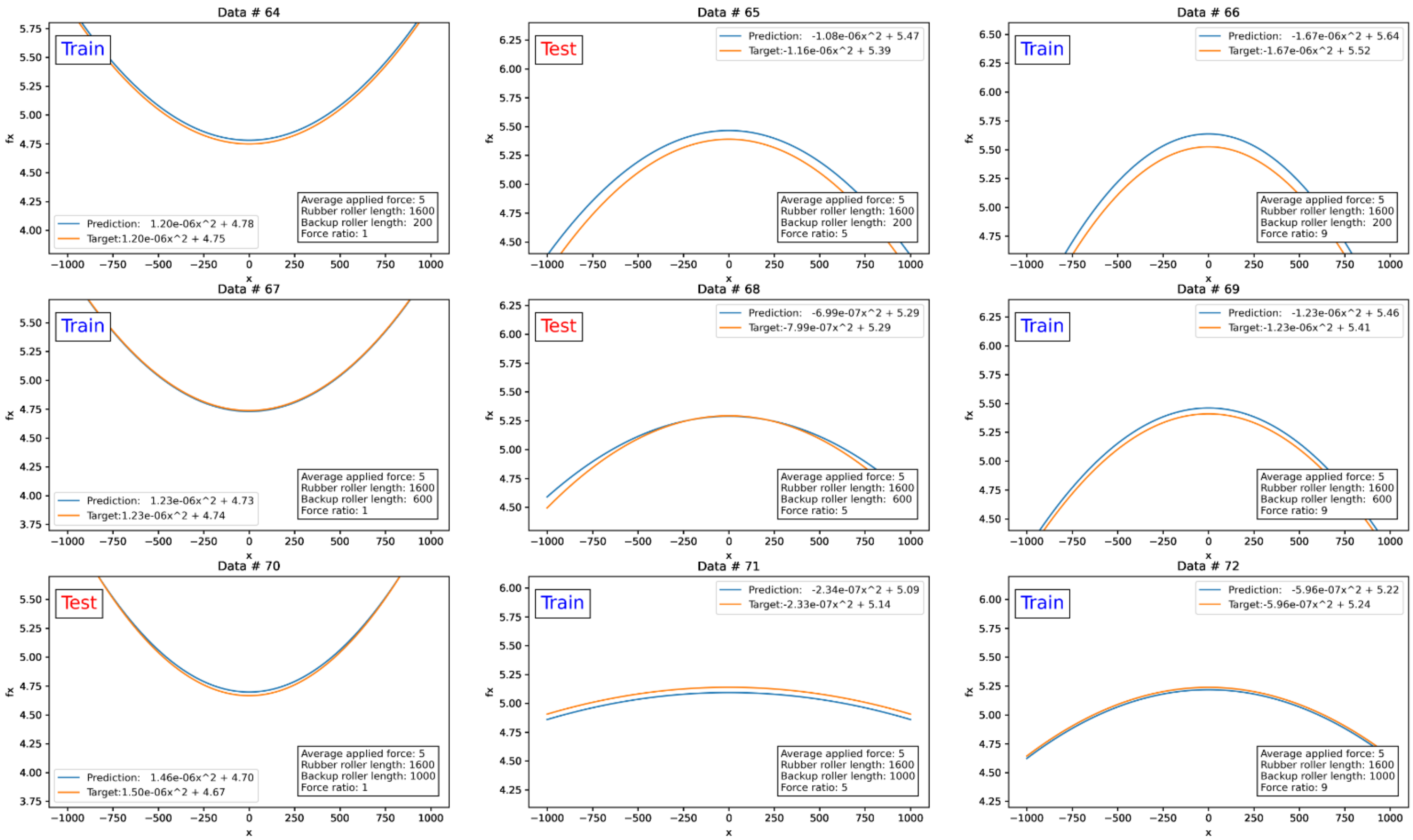
Data # 64
Train
Prediction: 1.20e-06x^2 + 4.78
Target:1.20e-06x^2 + 4.75
Average applied force: 5
Rubber roller length: 1600
Backup roller length: 200
Force ratio: 1
Data # 65
Test
Prediction: -1.08e-06x^2 + 5.47
Target:-1.16e-06x^2 + 5.39
Average applied force: 5
Rubber roller length: 1600
Backup roller length: 200
Force ratio: 5
Data # 66
Train
Prediction: -1.67e-06x^2 + 5.64
Target:-1.67e-06x^2 + 5.52
Average applied force: 5
Rubber roller length: 1600
Backup roller length: 200
Force ratio: 9
Data # 67
Train
Prediction: 1.23e-06x^2 + 4.73
Target:1.23e-06x^2 + 4.74
Average applied force: 5
Rubber roller length: 1600
Backup roller length: 600
Force ratio: 1
Data # 68
Test
Prediction: -6.99e-07x^2 + 5.29
Target:-7.99e-07x^2 + 5.29
Average applied force: 5
Rubber roller length: 1600
Backup roller length: 600
Force ratio: 5
Data # 69
Train
Prediction: -1.23e-06x^2 + 5.46
Target:-1.23e-06x^2 + 5.41
Average applied force: 5
Rubber roller length: 1600
Backup roller length: 600
Force ratio: 9
Data # 70
Test
Prediction: 1.46e-06x^2 + 4.70
Target:1.50e-06x^2 + 4.67
Average applied force: 5
Rubber roller length: 1600
Backup roller length: 1000
Force ratio: 1
Data # 71
Train
Prediction: -2.34e-07x^2 + 5.09
Target:-2.33e-07x^2 + 5.14
Average applied force: 5
Rubber roller length: 1600
Backup roller length: 1000
Force ratio: 5
Data # 72
Train
Prediction: -5.96e-07x^2 + 5.22
Target:-5.96e-07x^2 + 5.24
Average applied force: 5
Rubber roller length: 1600
Backup roller length: 1000
Force ratio: 9
fx
x

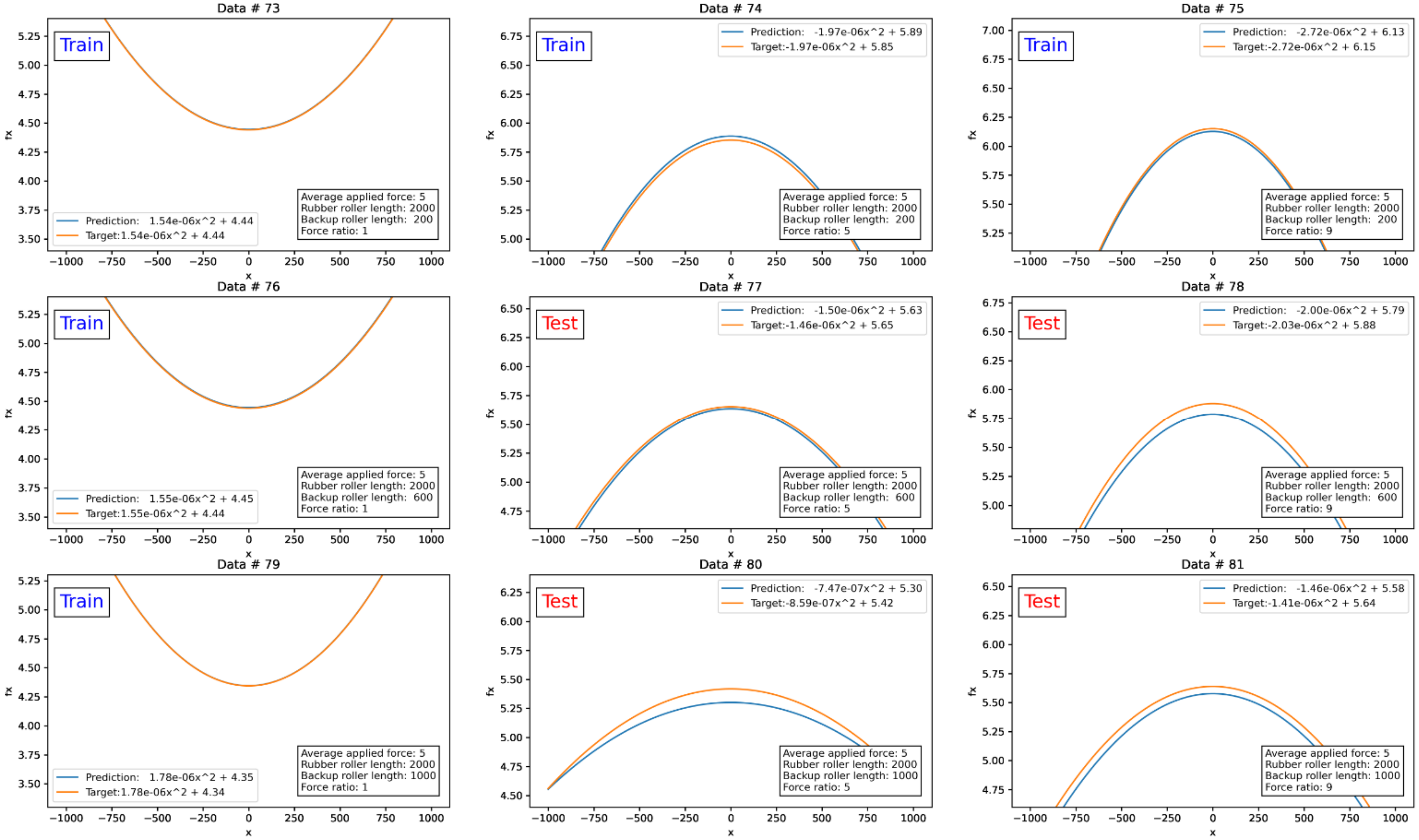
Data # 73
Train
Prediction: 1.54e-06x^2 + 4.44
Target:1.54e-06x^2 + 4.44
Average applied force: 5
Rubber roller length: 2000
Backup roller length: 200
Force ratio: 1
Data # 74
Train
Prediction: -1.97e-06x^2 + 5.89
Target:-1.97e-06x^2 + 5.85
Average applied force: 5
Rubber roller length: 2000
Backup roller length: 200
Force ratio: 5
Data # 75
Train
Prediction: -2.72e-06x^2 + 6.13
Target:-2.72e-06x^2 + 6.15
Average applied force: 5
Rubber roller length: 2000
Backup roller length: 200
Force ratio: 9
Data # 76
Train
Prediction: 1.55e-06x^2 + 4.45
Target:1.55e-06x^2 + 4.44
Average applied force: 5
Rubber roller length: 2000
Backup roller length: 600
Force ratio: 1
Data # 77
Test
Prediction: -1.50e-06x^2 + 5.63
Target:-1.46e-06x^2 + 5.65
Average applied force: 5
Rubber roller length: 2000
Backup roller length: 600
Force ratio: 5
Data # 78
Test
Prediction: -2.00e-06x^2 + 5.79
Target:-2.03e-06x^2 + 5.88
Average applied force: 5
Rubber roller length: 2000
Backup roller length: 600
Force ratio: 9
Data # 79
Train
Prediction: 1.78e-06x^2 + 4.35
Target:1.78e-06x^2 + 4.34
Average applied force: 5
Rubber roller length: 2000
Backup roller length: 1000
Force ratio: 1
Data # 80
Test
Prediction: -7.47e-07x^2 + 5.30
Target:-8.59e-07x^2 + 5.42
Average applied force: 5
Rubber roller length: 2000
Backup roller length: 1000
Force ratio: 5
Data # 81
Test
Prediction: -1.46e-06x^2 + 5.58
Target:-1.41e-06x^2 + 5.64
Average applied force: 5
Rubber roller length: 2000
Backup roller length: 1000
Force ratio: 9
fx
x

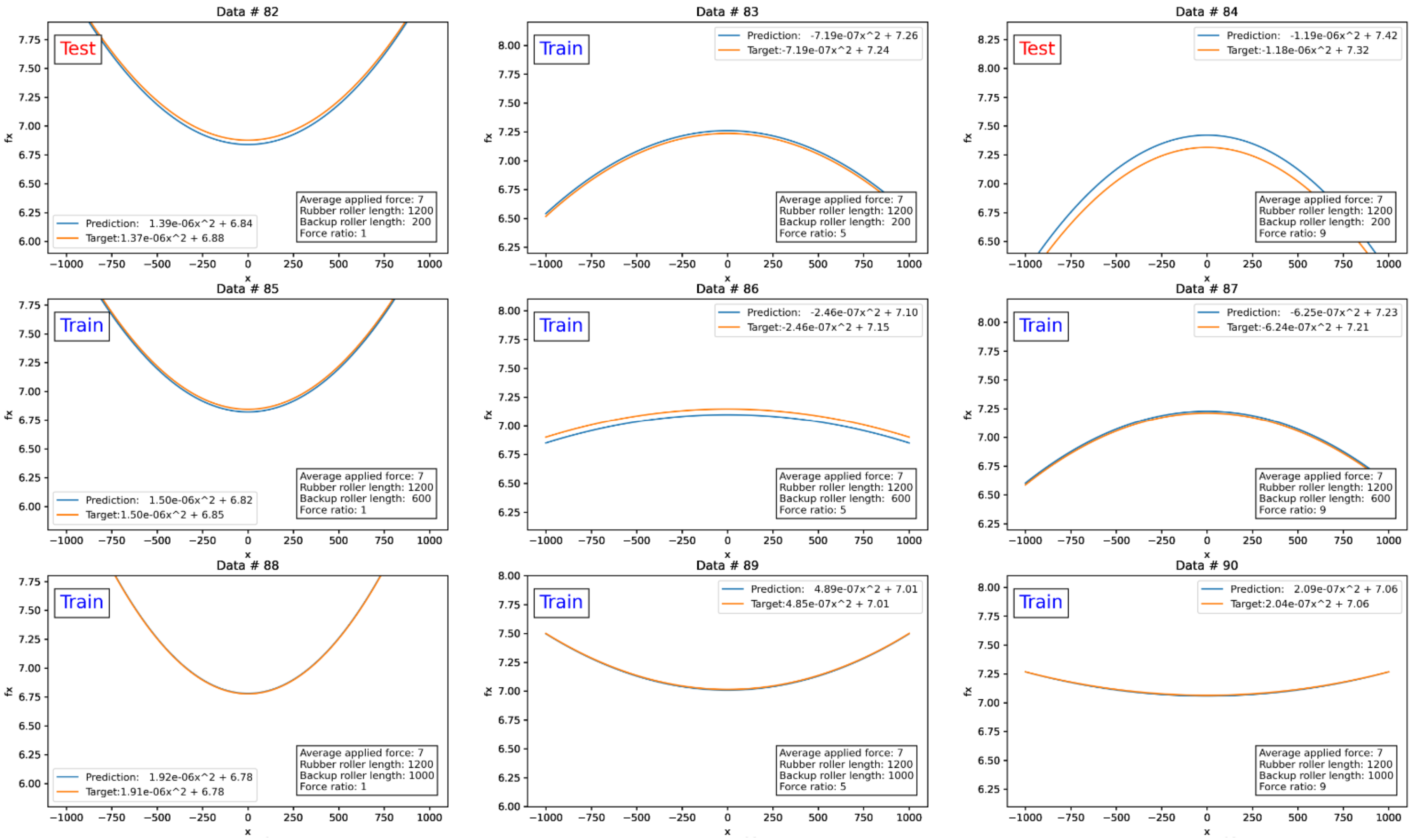
Data # 82
Test
Prediction: 1.39e-06x^2 + 6.84
Target:1.37e-06x^2 + 6.88
Average applied force: 7
Rubber roller length: 1200
Backup roller length: 200
Force ratio: 1
Data # 83
Train
Prediction: -7.19e-07x^2 + 7.26
Target:-7.19e-07x^2 + 7.24
Average applied force: 7
Rubber roller length: 1200
Backup roller length: 200
Force ratio: 5
Data # 84
Test
Prediction: -1.19e-06x^2 + 7.42
Target:-1.18e-06x^2 + 7.32
Average applied force: 7
Rubber roller length: 1200
Backup roller length: 200
Force ratio: 9
Data # 85
Train
Prediction: 1.50e-06x^2 + 6.82
Target:1.50e-06x^2 + 6.85
Average applied force: 7
Rubber roller length: 1200
Backup roller length: 600
Force ratio: 1
Data # 86
Train
Prediction: -2.46e-07x^2 + 7.10
Target:-2.46e-07x^2 + 7.15
Average applied force: 7
Rubber roller length: 1200
Backup roller length: 600
Force ratio: 5
Data # 87
Train
Prediction: -6.25e-07x^2 + 7.23
Target:-6.24e-07x^2 + 7.21
Average applied force: 7
Rubber roller length: 1200
Backup roller length: 600
Force ratio: 9
Data # 88
Train
Prediction: 1.92e-06x^2 + 6.78
Target:1.91e-06x^2 + 6.78
Average applied force: 7
Rubber roller length: 1200
Backup roller length: 1000
Force ratio: 1
Data # 89
Train
Prediction: 4.89e-07x^2 + 7.01
Target:4.85e-07x^2 + 7.01
Average applied force: 7
Rubber roller length: 1200
Backup roller length: 1000
Force ratio: 5
Data # 90
Train
Prediction: 2.09e-07x^2 + 7.06
Target:2.04e-07x^2 + 7.06
Average applied force: 7
Rubber roller length: 1200
Backup roller length: 1000
Force ratio: 9
fx
x

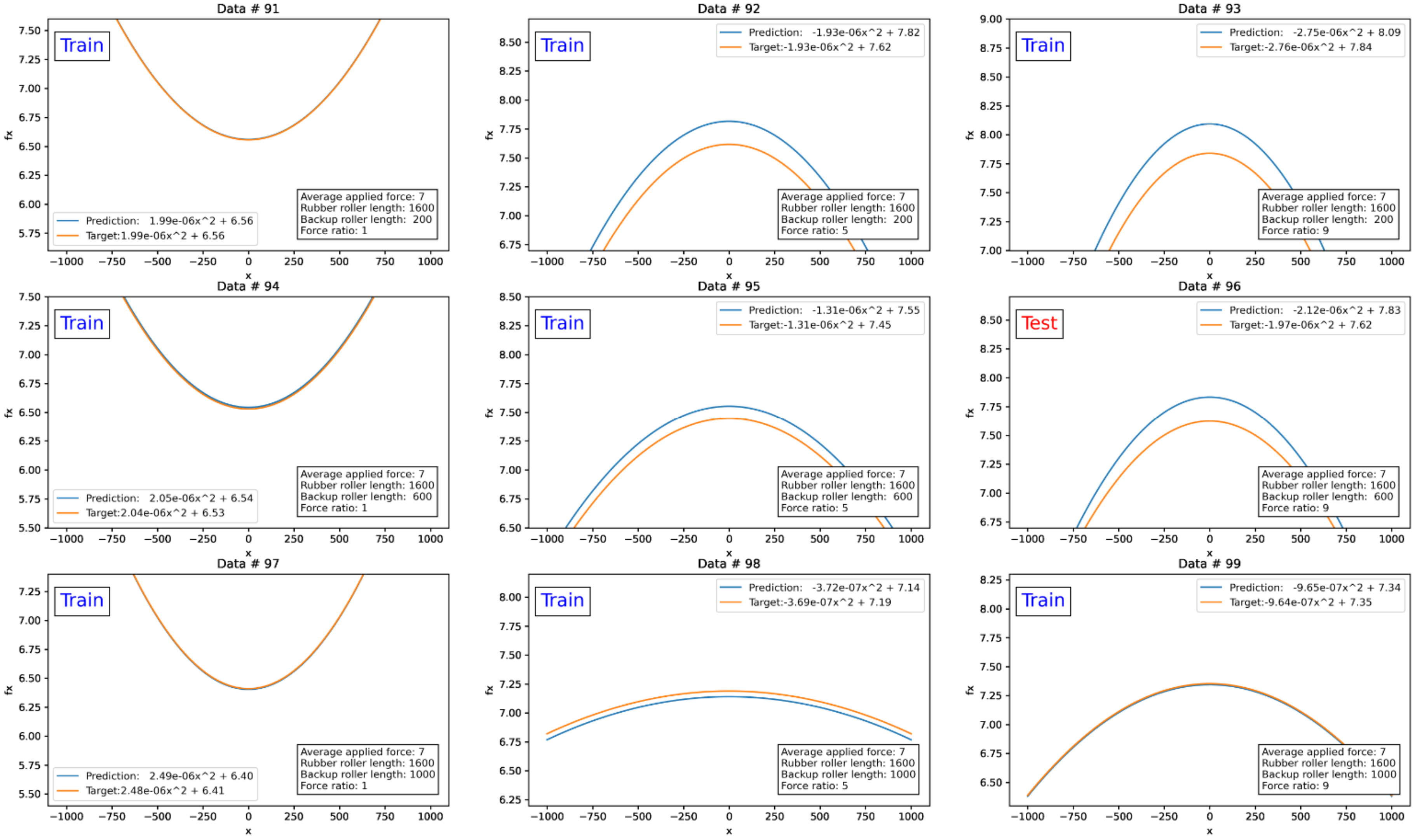
Data # 91
Train
Prediction: 1.99e-06x^2 + 6.56
Target:1.99e-06x^2 + 6.56
Average applied force: 7
Rubber roller length: 1600
Backup roller length: 200
Force ratio: 1
Data # 92
Train
Prediction: -1.93e-06x^2 + 7.82
Target:-1.93e-06x^2 + 7.62
Average applied force: 7
Rubber roller length: 1600
Backup roller length: 200
Force ratio: 5
Data # 93
Train
Prediction: -2.75e-06x^2 + 8.09
Target:-2.76e-06x^2 + 7.84
Average applied force: 7
Rubber roller length: 1600
Backup roller length: 200
Force ratio: 9
Data # 94
Train
Prediction: 2.05e-06x^2 + 6.54
Target:2.04e-06x^2 + 6.53
Average applied force: 7
Rubber roller length: 1600
Backup roller length: 600
Force ratio: 1
Data # 95
Train
Prediction: -1.31e-06x^2 + 7.55
Target:-1.31e-06x^2 + 7.45
Average applied force: 7
Rubber roller length: 1600
Backup roller length: 600
Force ratio: 5
Data # 96
Test
Prediction: -2.12e-06x^2 + 7.83
Target:-1.97e-06x^2 + 7.62
Average applied force: 7
Rubber roller length: 1600
Backup roller length: 600
Force ratio: 9
Data # 97
Train
Prediction: 2.49e-06x^2 + 6.40
Target:2.48e-06x^2 + 6.41
Average applied force: 7
Rubber roller length: 1600
Backup roller length: 1000
Force ratio: 1
Data # 98
Train
Prediction: -3.72e-07x^2 + 7.14
Target:-3.69e-07x^2 + 7.19
Average applied force: 7
Rubber roller length: 1600
Backup roller length: 1000
Force ratio: 5
Data # 99
Train
Prediction: -9.65e-07x^2 + 7.34
Target:-9.64e-07x^2 + 7.35
Average applied force: 7
Rubber roller length: 1600
Backup roller length: 1000
Force ratio: 9
fx
x

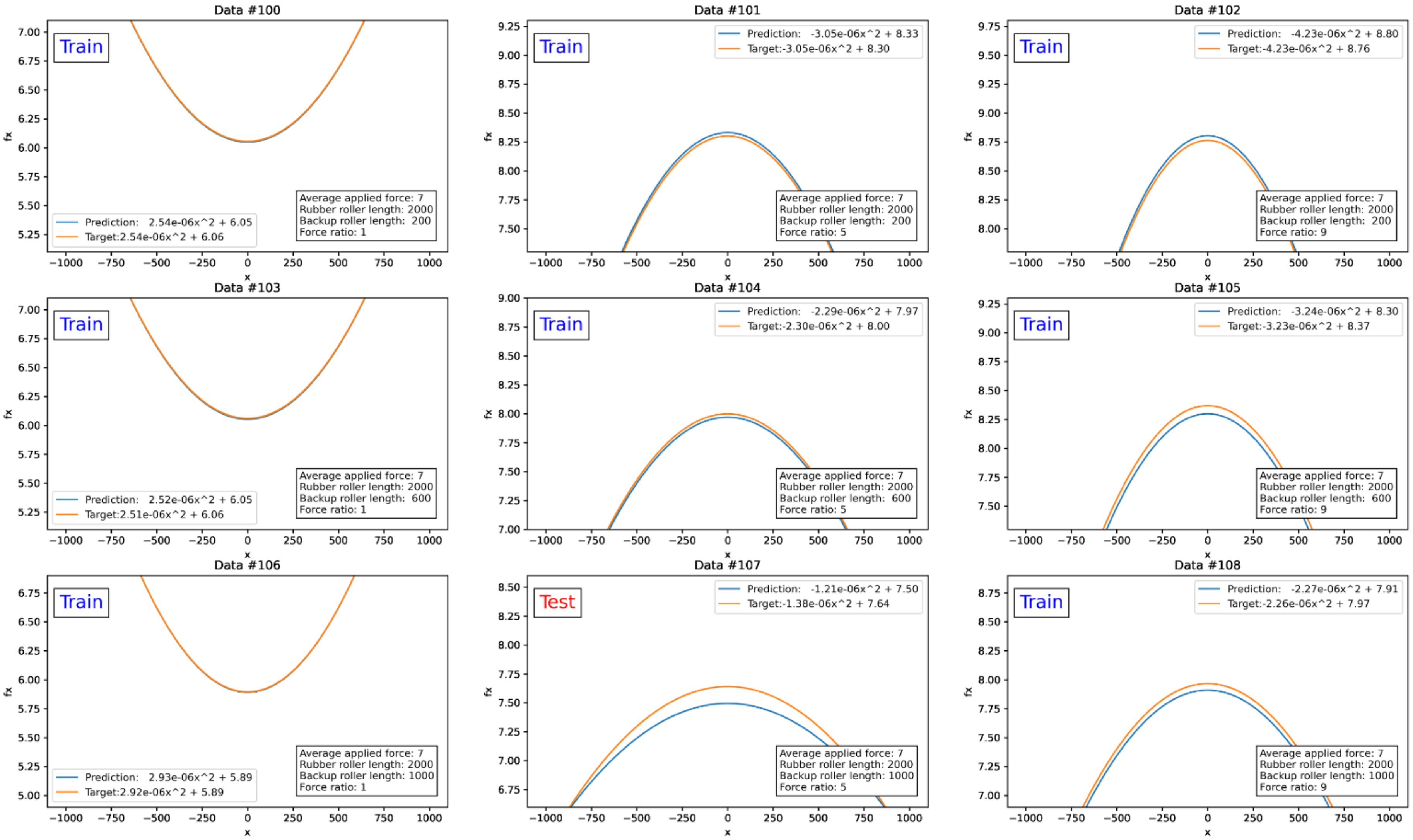
Data #100
Train
Prediction: 2.54e-06x^2 + 6.05
Target:2.54e-06x^2 + 6.06
Average applied force: 7
Rubber roller length: 2000
Backup roller length: 200
Force ratio: 1
Data #101
Train
Prediction: -3.05e-06x^2 + 8.33
Target:-3.05e-06x^2 + 8.30
Average applied force: 7
Rubber roller length: 2000
Backup roller length: 200
Force ratio: 5
Data #102
Train
Prediction: -4.23e-06x^2 + 8.80
Target:-4.23e-06x^2 + 8.76
Average applied force: 7
Rubber roller length: 2000
Backup roller length: 200
Force ratio: 9
Data #103
Train
Prediction: 2.52e-06x^2 + 6.05
Target:2.51e-06x^2 + 6.06
Average applied force: 7
Rubber roller length: 2000
Backup roller length: 600
Force ratio: 1
Data #104
Train
Prediction: -2.29e-06x^2 + 7.97
Target:-2.30e-06x^2 + 8.00
Average applied force: 7
Rubber roller length: 2000
Backup roller length: 600
Force ratio: 5
Data #105
Train
Prediction: -3.24e-06x^2 + 8.30
Target:-3.23e-06x^2 + 8.37
Average applied force: 7
Rubber roller length: 2000
Backup roller length: 600
Force ratio: 9
Data #106
Train
Prediction: 2.93e-06x^2 + 5.89
Target:2.92e-06x^2 + 5.89
Average applied force: 7
Rubber roller length: 2000
Backup roller length: 1000
Force ratio: 1
Data #107
Test
Prediction: -1.21e-06x^2 + 7.50
Target:-1.38e-06x^2 + 7.64
Average applied force: 7
Rubber roller length: 2000
Backup roller length: 1000
Force ratio: 5
Data #108
Train
Prediction: -2.27e-06x^2 + 7.91
Target:-2.26e-06x^2 + 7.97
Average applied force: 7
Rubber roller length: 2000
Backup roller length: 1000
Force ratio: 9
x
fx
